# Supplementary material for: Parental relatedness through time revealed by runs of homozygosity in ancient DNA
Source: Nat Commun. 2021 Sep 14;12:5425. doi: 10.1038/s41467-021-25289-w (PMC8440622; doi:10.1038/s41467-021-25289-w)
Supplement: Supplementary file 1 — Supplementary Information [file 41467_2021_25289_MOESM1_ESM.pdf]

Supplementary Information:
Human parental relatedness through time revealed by runs of homozygosity in ancient DNA

Harald Ringbauer[1,2,†], John Novembre[2,3,*] and Matthias Steinrücken[2,3,*]

[1]Department of Archaeogenetics, Max Planck Institute for Evolutionary Anthropology, Leipzig, Germany
[2]Department of Human Genetics, University of Chicago, Chicago, IL 60637, USA.
[3]Department of Ecology and Evolution, University of Chicago, Chicago, IL 60637, USA.
*These authors contributed equally to this work.
†Corresponding author – Email: harald_ringbauer@eva.mpg.de

June 2021

Supplementary Note 1 The hidden Markov model

We first describe how to model diploid genotype data y from a focal individual and a reference panel of n phased haplotypes $x_1, \ldots, x_n$ at a set of L loci, assuming biallelic markers. Thus, $y \in \{0, 1, 2\}^L$ and $x_i \in \{0, 1\}^L$. In Supplementary Note 1.3, we describe how types of data y relevant to applications using low-coverage sequencing data, like ancient DNA, can be modeled by treating the unobserved diploid genotypes as latent variables and using appropriate emission probabilities.

Throughout, we measure the distance between loci along haplotypes in genetic map units (i.e. Morgans) $\mathbf{r} = r_1, \ldots, r_{L-1}$, where r_l denotes the distance between locus $l+1$ and l. We assume that a genetic map is available, which is the typical case for humans and model organisms. If no genetic map is available, the map distances can be approximated using the average recombination rate, but we note that here we only tested scenarios where a map is available.

Supplementary Note 1.1 State Space

The Hidden Markov model (HMM) can assume any of $n+1$ hidden states $0, \ldots, n$ at every marker l, where n is the number of haplotypes in the reference panel. As we outline below, the 0-th state represents that the focal individual is not in a ROH at the respective marker, and has emission probabilities according to Hardy-Weinberg proportions, while the states $1, \ldots, n$ are the classical copying states (Fig. S1). In each of these copying states (denoted here as the ROH states), we model the copying as in the original Li & Stephens model [1], with one important modification: We assume that the genotype of the focal individual y

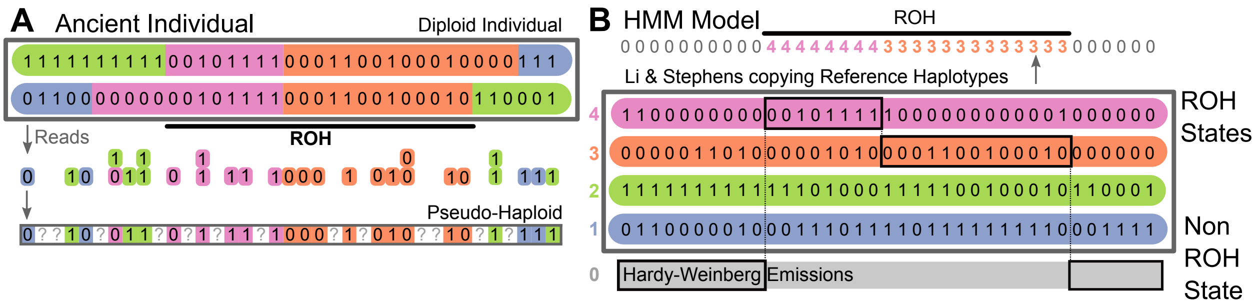

Figure S1: Detecting runs of homozygosity using a reference panel. Panel A: Illustration of genotype data from a diploid individual. Sequencing reads mapping to a biallelic SNP produces counts of reads for each allele, from which in turn pseudo-haplotype genotypes, i.e. single reads per site, are sampled (at random). Panel B: Schematic of Method. A target individuals genotype data is modeled as being copied from a reference panel (colored) and one additional non-ROH state, where copying probabilities are given by Hardy-Weinberg proportions.

is homozygous for the allele of the reference haplotype that it copies from. The emission probabilities are specific to the exact kind of data that is analyzed, and can include various types of error models, which we discuss in Supplementary Note 1.3.

In the Hardy-Weinberg state 0, the probabilities of observing a diploid genotype reflect the probabilities of an underlying genotype in Hardy-Weinberg equilibrium, with probabilities of the alleles according to the underlying allele frequency in the reference panel at this locus. We note this state is identical to the non-ROH state used in a previously developed HMM to call ROH [2].

Supplementary Note 1.2 Infinitesimal Transition Rates

To define a hidden Markov model, one needs to specify the transition probabilities between the hidden states for each pair of successive loci l and $l+1$. In our model, we do so by using an infinitesimal rate matrix Q of dimension $(n+1) \times (n+1)$, from which the transition probability matrix $A_{l \to l+1}$ can be obtained via exponentiation: $A_{l \to l+1} = \exp(Q \cdot r_l)$, where r_l is the genetic distance between the respective loci.

As in the Li & Stephens copying model [1], every reference haplotype $i = 1, \ldots, n$ is equally likely to be copied from. Thus the transition probabilities between these copying states (copying a homozygous genotype from the respective reference haplotype) are symmetric, and the transition rates to and from a copying state $i = 1, \ldots, n$ do not depend on the reference haplotype i. We can thus specify the infinitesimal rate matrix by three parameters: A single rate for the transition from the non-copying into a copying state Q_{0j} for all $j > 0$, a single rate for leaving a copying state Q_{j0} for all $j > 0$ and a third rate for transitioning from one copying state to another $\phi_{\text{ROH}} = Q_{jk}$ for all $j, k > 0, j \neq k$. The diagonal entries of the rate matrix Q are determined by the rate matrix condition $Q_{ii} = -\sum_{j \neq i} Q_{ij}$.

We point out that in the limit of infinite jumping rates within ROH ($\phi_{\text{ROH}} \to \infty$), our model converges to the full model of [2], as the probabilities of being in one of the allelic states (the sum of probabilities of copying from all reference haplotypes that have this allelic

state) will then reflect its frequency, as in this limit jumps occur between any two consecutive markers.

Supplementary Note 1.3 Emission Probabilities

In our model, the emission probabilities that specify the probability of observing the data at locus l given some hidden state i, $e_i(y_l)$ depend on the type of data. We implemented two emission models that we applied for analysing empirical data: diploid genotype data and pseudo-haploid genotype data, with both of them incorporating a model for genotype error. Throughout, we always disregard markers with missing data by removing them both from the reference panel as well as the target and adjusting the transition rates accordingly.

We implemented the emission model for diploid genotypes as follows. In the non-ROH state (i=0), the Hardy-Weinberg emission probabilities for the genotypes are $(1-p_l)^2$, $2p_l(1-p_l)$, and p_l^2, for observing homozygosity for the ancestral allele, heterozygosity, and homozygosity for the derived allele, respectively, where p_l is the frequency of the derived allele in the reference panel at locus l. For the ROH-states ($i = 1, \ldots, n$), the genotype probabilities are 1 to be homozygous for the allelic type of the source haplotype in the reference panel, and 0 for the two other possible diploid genotypes. We extend these genotype probabilities to model possibly erroneous genotypes: A genotype is homozygous for the copied allele with probability $1-\epsilon$ and it is flipped to one of the two other possible genotypes with probability $\epsilon/2$ respectively. This simplified error model has the advantage of having only a single parameter while broadly modeling a wide range of possible errors, including genotyping error in the reference as well as in the target, or new mutations that are private to the target individual. We note that for ancient DNA data, where genotyping error rates (including errors due to contamination) are typically on the order of $10^{-2} - 10^{-3}$ [3], the genotyping error rate will be the main driver of ϵ, as for modern human populations the reference panel is almost always separated no more than 10^5 generations from the target. The per base-pair mutation rate is on the order of 10^{-8} per generation, which results in an upper bound for the substitution rate of order 10^{-3}.

The second emission model we implemented is for pseudo-haploid genotype data, a common data type for human ancient DNA. An observed pseudo-haploid genotype can have two states (ancestral or derived), and the emission probabilities for the two possible observations are given as follows. For the copying states ($i = 1, \ldots, n$), the allele on haplotype i is emitted with probability $1-\epsilon$, and the other allele is emitted with probability ϵ. For the non-ROH state (i=0), the emission probabilities model sampling one read from an underlying genotype in Hardy-Weinberg equilibrium under the allele frequencies in the reference panel: A derived pseudo-haploid marker is observed with probability p_l, and an ancestral marker with probability $1-p_l$. To account for errors, with probability ϵ the observed read actually reflects the opposite allelic state. As in the case of diploid genotypes, this error rate ϵ models both the disagreement rate due to new mutations occurring on the genealogical lineage between the reference haplotype and the target, as well as the rate of genotyping errors.

We also implemented an experimental third emission model designed for read count data simulated under an idealized model. Here, the data for a specific locus consists of n reads, with k of them mapping to the derived allele and $n-k$ to the reference allele. Given the underlying genotype, modeled probabilistically as in the diploid genotype case described

above, we add a second layer that describes the sampling of the n reads. We use a binomial likelihood model, where the probability of observing k out n reads carrying the derived allele is binomial with probability $p = 0$, $p = 0.5$, and $p = 1$ given the heterozygous ancestral, homozygous, and heterozygous derived genotype, respectively. We add two levels of error: One at the read level, where each read is flipped to the opposite allele with probability ϵ. We add an additional level of error at the genotype level, corresponding to the error model of erroneous diploid genotypes described above, where a diploid genotype is flipped to one of the other two possibilities with probability $\epsilon_{ref}/2$. This is to account for rare errors in the reference panel that would induce mismatches between the target individual's genotype.

We did not apply this last model on empirical ancient DNA data because the assumption of ancient DNA data being modeled well by a binomial likelihood of read counts is likely often violated for such data. In principal, extensions for more complex models could be incorporated by adjusting the emission probabilities linking the unobserved diploid genotypes to the data and any future extensions could be naturally modeled using a genotype likelihood framework that describes the likelihood of the observed data under each of the three possible latent diploid genotype states. However, obtaining appropriate genotype likelihoods for ancient DNA is a topic of much ongoing research [e.g. 4], as accurate genotype likelihoods rely on non-trivial models (modeling reference bias, interdependence of reads, genotype errors) that in turn depend on the type of ancient DNA data generated (e.g. shotgun or capture data, UDG treatment, length of sequencing reads, single versus double stranded sequencing). Therefore, we decided to use pseudo-haploid data in our empirical application as such data requires fewer assumptions. While in principle perfect genotype likelihoods contain more information than pseudo-haploid data, based on simulated data we expect to see only small differences when analyzing low-coverage data (Fig. S5). The reason is that at low coverage the majority of sites are only covered by at most one read, and thus the pseudo-haploid genotype data contains most of the available information already.

Supplementary Note 1.4 Posterior Decoding

We use standard Hidden Markov model algorithms to calculate the posterior probability $P(\pi_l = i|y)$ of the hidden states $i = 0, 1 \ldots, n$ at locus l observing the data $y_1, \ldots y_L$ [5]. Specifically, we compute the forward probabilities,

$$f_i(l) := P(y_1, \ldots, y_l, \pi_l = i) = e_i(y_l) \sum_{k=0}^{n} f_k(l-1) A_{ki}, \tag{1}$$

as well as the backward probabilities,

$$b_k(l) := P(y_{l+1}, \ldots, y_L | \pi_l = k) = \sum_{i=0}^{n} A_{ki} e_i(x_{l+1}) b_i(l+1), \tag{2}$$

using dynamic programming, where A denotes the transition matrix $A_{l-1 \to l}$. Together, these are combined to obtain the posterior:

$$P(\pi_l = i|y) = \frac{f_i(l) b_i(l)}{P(y)}, \tag{3}$$

where $P(y)$ denotes the full probability of the data, which can be computed as $P(y) = \sum_k f^n_{k=0}(L)$.

To complete the posterior decoding and thereby call ROH segments, we use posterior thresholding. We return consecutive regions where the posterior probability of the non-ROH state remains below the threshold $1 - T$, or equivalently the sum of the posteriors of the copy states is above T. In Supplementary Note 1.8 we describe the procedure for how we set the default value of T for our implementation of the method.

Supplementary Note 1.5 Computational Speedup

The runtime (and memory requirement) of the algorithm for the posterior decoding of the HMM scales linearly with the number of loci L that are analyzed. In the naive implementation, the scaling with the number of hidden states K (the number of reference haplotypes plus one here) is quadratic, since the full transition matrix has to be computed and each entry employed in Equation (1) and (2). Thus, the runtime of the naive implementation is $\mathcal{O}(LK^2)$.

However, as is standard for these models, we can reduce this runtime to linear in the number of hidden states, to $\mathcal{O}(LK)$, by using the symmetry of the copying states: For hidden state $i > 0$, the sum in Equation (1) can be split up into three parts (we suppress dependencies on $l - 1$ here):

$$\sum_k f_k A_{ki} = \underbrace{f_0 A_{0i}}_{I} + \underbrace{\sum_{k>0} f_k A_{12}}_{II} + \underbrace{f_i(A_{ii} - A_{12})}_{III}, \tag{4}$$

where we used that $A_{ki} = A_{12}$ for all $k, i > 0$, which follows from the symmetry of the transition rate matrix Q. Similarly, for k=0 we get:

$$\sum_k f_k A_{k0} = \underbrace{f_0 A_{00}}_{I} + \underbrace{\sum_{k>0} f_k A_{10}}_{II}, \tag{5}$$

because $A_{k0} = A_{10}$ for all $k > 0$.

The quadratic dependence of the runtime on the number of states is caused by the sum in II in Equation (4), and similarly in Equation (5). However, when updating the forward probabilities $f_i(l)$ for all states i, we only need to pre-compute $\sum_{k>0} f_k$ once for every locus. Doing so achieves the reduction to linear runtime. The backward algorithm can be modified analogously, with first splitting the sum in Equation (2) and then pre-computing $\sum_{i>0} A_{ki} e_i b_i$ only once when updating $b_k(l)$ for all states k.

Supplementary Note 1.6 Computing the transition matrices

In the naive implementation of our algorithm, the infinitesimal rate matrix Q has to be exponentiated at every locus l, which would be computationally costly (depending on the implementation scaling quadratic or worse with number of states). However, due to the speed-up described in Supplementary Note 1.5, we only require a small subset of the entries

of the full transition matrix, namely A_{00}, A_{11}, A_{12}, A_{01} and A_{10}. We note that a truly symmetric model (such as the original Li & Stephens copying model) could be reduced even further into a single transition rate [the probability of staying in a copy state, 6]. However, due to the additional non-ROH state here, one has to keep track of at least three rates, and these can be efficiently pre-compute as follows.

Using the symmetry of the copying states $1, \ldots, n$, we can collapse these states into state 1 and a single surrogate state for $2, \ldots, n$. We then only need to consider the states 0,1, and the surrogate state, thus arriving at a 3×3 transition rate matrix $\tilde{Q}$, where $\tilde{Q}_{ij} = Q_{ij}$ for $i \leqslant 2$, $j < 2$ and $\tilde{Q}_{i2} = \sum_{j>1} Q_{ij} = (n-1)Q_{i2}$ for $i < 2$. Importantly, by exponentiation of $\tilde{Q}$ the three relevant entries of A can be recovered by first computing $\tilde{A} = \exp(\tilde{Q})$ and then using $A_{ij} = \tilde{A}_{ij}$ for $i, j < 2$ and $A_{12} = \tilde{A}_{12}/(n-1)$.

To efficiently incorporate variable recombination distances between loci, we first diagonalize the common collapsed rate matrix: $\tilde{Q} = P^{-1}\tilde{D}P$. For each locus l, we can then exponentiate using $\exp(\tilde{Q} \cdot r) = P^{-1}\exp(\tilde{D} \cdot r_l)P$, which only requires exponentiation of a diagonal matrix, and recover the corresponding entries of $\tilde{A}$ and consequently A required for calculating the full posterior. In Supplementary Note 1.8 we describe the procedure for how we set the default rates of Q for our implementation.

Supplementary Note 1.7 Simulating Genetic Data with ROH

To test the performance of our method, we simulated genetic data with known ROH. We use this data below to carry out experiments where we down-sample to lower coverage and add genotyping errors to 1) help determining robust HMM parameters (Supplementary Note 1.8) and to 2) test the performance (Supplementary Note 1.8). First, we describe the method we used to generate these simulated datasets with known ROH.

We used a copying approach inspired by [7] to generate ground-truth ROH block sharing data for testing methods. A synthetic mosaic individual without long ROH >1 cM is first generated by concatenating stretches of diploid genotypes in 0.25 cM tracts from randomly chosen individuals of the reference set. The intuition is that the probability of long ROH blocks (>1 cM) arising inadvertently is very low (as multiple ROH blocks would have to be concatenated), while still mostly retaining local LD structure typical for diploid human individuals. In our simulations, we used the positions of a widely used set of 1.24 million SNPs widely used for human ancient DNA studies [1240K capture technology for 8], and we focused on chromosome 3, a human chromosome with a typical density of these sites per map unit (Morgan).

We then copied in five ROH blocks of a given length uniformly at random, enforcing that ROH blocks do not overlap by placing them at random in 5 evenly split up sectors of the chromosome. The copied-in stretch originates from one haplotype of the source population (chosen uniformly), and both alleles of the synthetic individual are set to the allele of the copied-in stretch. The source population for the simulations is then excluded from the reference panel. These synthetic mosaic individuals, with known diploid genotypes, serve as test cases for the method. These data were down-sampled and error added to it to simulate data of varying quality (e.g. Fig. S2A,B).

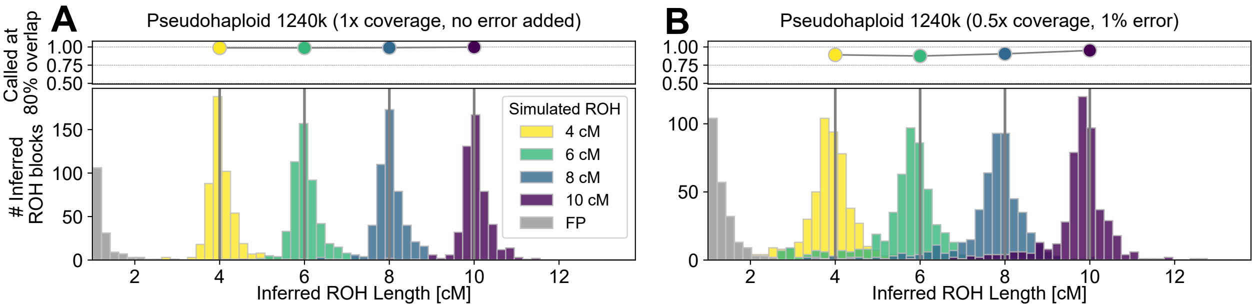

Figure S2: Detecting simulated ROH. Panel A: We applied our method to simulated data with known ROH copied. We copied in ROH of either 4, 6, 8, and 10 cM length (5 of every length class into each of 100 simulated chromosomes, Supplementary Note 1.7), and depict histograms of inferred ROH lengths (in color) as well as false positives (in gray). Panel B: Same as panel A, but a simulation with erroneous and missing data typical for lower quality ancient DNA data.

Supplementary Note 1.8 Choosing Parameters

The model has several parameters that have to be set when analyzing data. Here we describe how we set the default parameters we used throughout our empirical analysis and our simulation experiments (unless explicitly stated otherwise). We set the infinitesimal transition rates based on the typical tracts we are interested to find. Our target use case here is to detect ROH blocks that are of length 5 cM that occur once every 100 cM. Accordingly, we chose the infinitesimal rate parameters (per Morgan) as 1 (jump from non-ROH into ROH) and 20 (jump from a ROH state into non-ROH). We fixed the transition rate between ROH states (i.e. the haplotype copying model switch rate) to 300 per Morgan, corresponding to an average copy tract length of ca. 0.3 cM. This value was chosen based on performance of ROH calling in pilot simulations and a likelihood profile of a Li & Stephens model of Tuscany haplotypes from all non-Tuscany Europeans in the 1000 Genomes dataset.

The emission probabilities described above (Supplementary Note 1.3) contain an error rate parameter ϵ. Throughout our analysis and for the determination and further evaluation of parameters, we set $\epsilon = 0.01$. This error rate is a representative use case for our method: Error rates cover sequencing errors, ancient DNA damage, both typically not exceeding 1% [9, 10] and contamination (mostly below 5%, Fig. S19, and not all contamination results in erroneous reads). Below, we explore how various rates of genotype error behave when using the default error rate in the HMM, showing that our goal to set an error rate that works robustly in a wide range of scenarios (from no error to error rates of a few percent) was achieved (Fig. S4).

To determine a robust posterior threshold, we ran simulation experiments with data typical for our use case, which is analysis of 1240K pseudo-haploid data with the full 1000 Genomes dataset set as a reference panel. As test cases, we simulated mosaics of chromosome 3 with pseudo-haploid data, i.e. one allele chosen at random, and then down-sampled (at random) to 50% of all 1240K SNPs covered (and the rest set as missing data). We then flipped the allele with probability 0.01 to the other allele to simulate data with low quality.

The reason for choosing the cutoff based on low quality data is that we want the cutoff

to be robust in these cases. We tradeoff maximum specificity for high quality data (where more aggressive cutoff settings would be possible) to allow our method being applicable to a wide range of use cases with default parameters.

Using the TSI (Tuscany, Italy) samples from the 1000 Genomes dataset, we simulated 100 replicates of mosaics of chromosome 3 for two scenarios: 1) with 4 cM ROH blocks copied in (to determine power and bias of inferred ROH length) 2) no blocks copied in as well (to assess false positives). We then ran the method using the 1000 Genomes dataset and only TSI individuals removed as reference panel, tested various posterior cutoffs, and monitored false positive rate, power, length bias, and standard deviation of the longest block overlapping the true ROH blocks, with blocks of length 4 cM as the test case. When analyzing 100 replicates with various posterior cutoffs, we found that a cutoff of 0.998 lead to a good performance in terms of the magnitude of bias for ROH, as well as standard deviation of inferred length of ROH (Table S1). As our overall goal is to call ROH with little bias and also with little variation in length, we focused on minimizing standard deviation and bias, and chose a value of 0.998 as posterior cutoff in our implementation.

Posterior Cutoff	Rep.	STD 4cM	FP ROH>1cM	FP ROH>2cM	Avg. Bias 4 cM [cM]	Frac. 80% of 4 cM called
0.9	20	0.69	16.1	2.95	0.57	1.00
0.99	20	0.59	7.6	0.65	0.23	0.97
0.996	100	0.61	5.39	0.47	0.06	0.958
0.997	100	0.59	4.70	0.35	0.02	0.950
**0.998**	100	**0.57**	3.78	0.21	**-0.03**	0.930
0.999	100	0.60	2.34	0.11	-0.15	0.892
0.996	100	0.61	5.39	0.47	0.06	0.96
0.9999	20	0.97	0.2	0.00	-1.30	0.35
0.99999	20	0.85	0.0	0.00	-3.22	0.00

Table S1: Varying the posterior cutoff on various performance metrics. We varied the posterior cutoff used for calling ROH, calculated several summary statistics when calling ROH for mosaic individuals (TSI). For each line, 20 replicates for chromosome 3 with five 4 cM ROH copied or no ROH copied in were simulated to calculate the performance statistics. False positive rates (FP) are calculated as the average number of falsely inferred blocks per replicate chromosome. Standard deviation ("STD") is calculated from all inferred blocks overlapping the spiked in blocks. The column "bias" describes the average difference to 4 cM. In the parameter range with better performance, we added 80 replicates to improve the accuracy of the estimates. We chose 0.998 as threshold because it minimizes standard deviation and bias.

For applications on 1240K pseudo-haploid SNPs with at least 400,000 autosomal SNPs covered and using the 1000 Genomes data as the reference panel, this set of parameters can be readily applied, and we provide these parameters as the default settings in our software package that implements the method. For users who wish to apply our method to another set of SNPs, a different reference panel, or non-human data, we strongly recommend to repeat a similar strategy to find a suitable threshold in the respective scenario.

Despite intensive testing, we could not identify any set of parameters where 2 cM ROH was called robustly with an acceptable false positive rate for ROH of this length. We explored a wide range of transition rates (including parameters tuned to target shorter ROH), but

could not identify any set of parameters which provided a substantial improvement for shorter blocks. In particular the many false positives that appear around 2 cM for low coverage individuals (Fig. S4) persisted. We believe one reason is areas of low SNP density, whose effects become more prominent at this length scale (Fig. S18). We believe little can be done to achieve sufficient resolution at an acceptable false positive rate in this regime when running a genome-wide analysis. However, a promising future approach beyond genome-wide screens for ROH could be focused screening on areas of high SNP density.

Supplementary Note 1.9 Merging of Gaps between ROH

Motivated by the observation that the vast majority of false positive ROH are shorter than 2 cM (Fig. S2), we only record ROH blocks >2 cM. We observed that long ROH are sometimes broken up by spurious gaps (Fig. S3 and manual inspection of blocks where the length was substantially underestimated), as similarly seen in methods that call long IBD blocks between individuals [11]. Such gaps may arise due to genotyping error, structural variation or very low SNP density. Following a standard procedure of IBD block calling [7] and of genomic feature annotation with HMMs [5], we decided to merge gaps, as experiments with lowering the posterior threshold or with decreasing the jump rate introduced a large surplus of additional false positives. To ensure that we do not merge two false positives (the false positive rate >2 cM is non-zero), we additionally require at least one of the merged blocks to be longer than 4 cM, and the gaps to be less than 0.5 cM in length. Fig. S3 shows that this procedure improves the performance substantially.

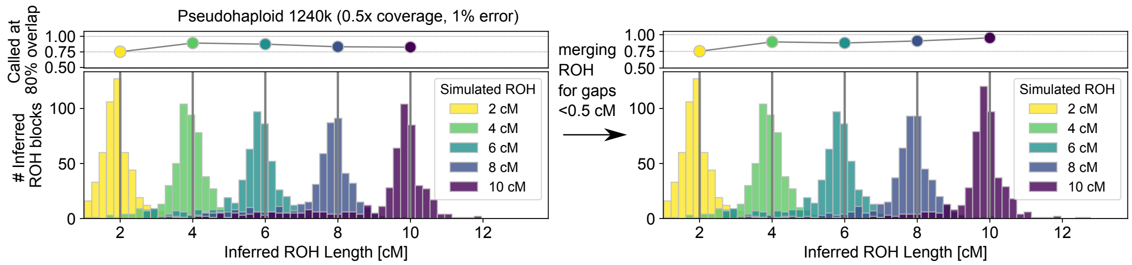

Figure S3: Improving power for long ROH blocks by merging gaps between ROH stretches We depict the effect of merging ROH gaps for the "worst case" simulation scenario where we expect our method to have the least power to detect uninterrupted segments of ROH. Merging gaps <0.5 cM for between blocks where the longer block >4 cM markedly improves performance for long ROH blocks (>8 cM), without changing the distribution of shorter ROH blocks (4 cM).

Supplementary Note 1.10 Runtime and Memory Requirements

The runtime of our algorithm in the implementation described above scales linearly with the number of haplotypes in the reference panel (see Supplementary Note 1.6) and linearly with the number of loci included in the analysis.

Similarly, the memory requirements scale linearly with the number of loci and linearly with the number of reference haplotypes. If memory requirements are an issue, one could

run the algorithm for overlapping segments along the genome. However, we did not hit such a limit in our applications. Thus, we ran the algorithm for data from whole chromosomes. We parallelized computations by running different chromosomes and different individuals simultaneously. This can be done by starting independent instances of the program for each target individual (which we did for the large empirical data analysis on a computational cluster). Moreover our program also has a parameter to run a specified number of threads simultaneously to analyze all chromosomes of a target individual, using the python library `multiprocessing`.

We report memory and runtime requirements of our algorithm (`hapROH` v0.3) using a single CPU of an Intel Xeon E5-2680 v4 (2.40GHz) processor. We ran tests using two ancient individuals: A high coverage individual and an individual with low coverage. As in our empirical analysis, we used pseudo-haploid data for all available autosomal 1240K SNPs and the full 1000 Genome reference data. For the high coverage individual (42x average depth, 1,147,829 autosomal SNPs with data), our algorithm uses up to 8,2 GB memory and finishes in approximately 850 seconds, with runtime per chromosome ranging from 22 to 75 seconds. Applying `hapROH` to the low coverage individual (I2534, Romania Mesolithic, 0.53x, 418,429 SNPs with data), our algorithm requires up to 2,9 GB memory and finishes in approximately 360 seconds, with runtime per chromosomes ranging from 17 to 38 seconds.

We note that these tests are only intended to give a general idea of the runtime and memory requirements. Future optimizations of the underlying code or the way the reference data is loaded and stored in memory could provide improvements over the runtime and memory requirements reported here.

Supplementary Note 2 Testing and Validation

Supplementary Note 2.1 Performance on simulated data

To test its performance, we applied our implementation of the method with default parameters chosen as described in Supplementary Note 1.8 to mosaic individuals with copied in ROH blocks as detailed in Supplementary Note 1.7. When applying the method to pseudo-haploid data down-sampled to varying degree, we found that it has high power (>95%) to detect ROH blocks >4 cM while having simultaneously a low false positive rate (Fig. S4A) down to ca. 0.3× covered 1240K sites. Moreover, we find that, when first applying random genotype errors, the method can tolerate genotype error rates up to 5% (Fig. S4B).

When downsampling to create pseudo-haploid data, each locus was kept with target probability p, and set to missing otherwise. We note that in ancient DNA studies applying 1240K capture, some loci have a systematically higher chance to be covered than others. However, our simple downsampling model should be a useful approximation as long as there are no population genetic biases affecting rates of missingness.

In our simulations, and also our modeling, we add genotype errors with equal probability to all sites, flipping the observed pseudo-haploid call to the other allele at random at a given rate ϵ. We note that in practice, errors are heterogeneous, e.g. C→T and G→A transitions are more prevalent in ancient DNA than transversions. The degree of heterogeneity depends on a plethora of technical details such as UDG treatment, the ancient DNA preservation, the position on each read and also what bioinformatic filtering has been applied. Instead of applying a complex error model, which will likely fail to capture the full complexity regardless, we chose to model and simulate data with a uniform error model. We assume that by using a slightly elevated uniform rate error for the whole set of SNPs, we can approximate the effects of unmodeled sources of error.

We also compared the performance of the pseudo-haploid emission mode (using one read per locus) and an experimental read count emission mode (using all reads per site). To this end, we simulated data with a variable number of reads per site according to a heterogeneous Poisson model using mosaic individuals as described in Supplementary Note 1.7. To mimic realistic read count distributions, which can be highly heterogeneous for 1240K capture data, we first calculated the ratio of total read depth per site and genome-wide read depth from a subset of empirical ancient DNA 1240K data with such data available [12] per site (calling these ratios λ_i, with i indexing sites). We then sampled at each SNP from a Poisson distribution with mean weighted by λ_i times the genome-wide coverage we wish to simulate. We then sample derived reads according to a binomial model with p =0, 0.5, or 1.0, depending on the underlying diploid genotype. In this simulation, we used average coverages of $0.1, 0.2, \ldots, 0.6\times$. We simulated 100 replicates using the mosaic chromosome 3 created from TSI (Tuscany) haplotypes described above and using the rest of the 1000 genome data as the reference panel.

Using this simulation strategy (which we term the "λ read count" model), the Poisson likelihood from Eq. (6) provides the exact genotype likelihood, therefore we compare pseudo-haploid emissions to the ideal genotype likelihoods. However, we find that in the low coverage regime typical for ancient DNA, there is only marginal improvement when using the exact likelihood model compared to using only pseudo-haploid emissions (Fig. S5). The

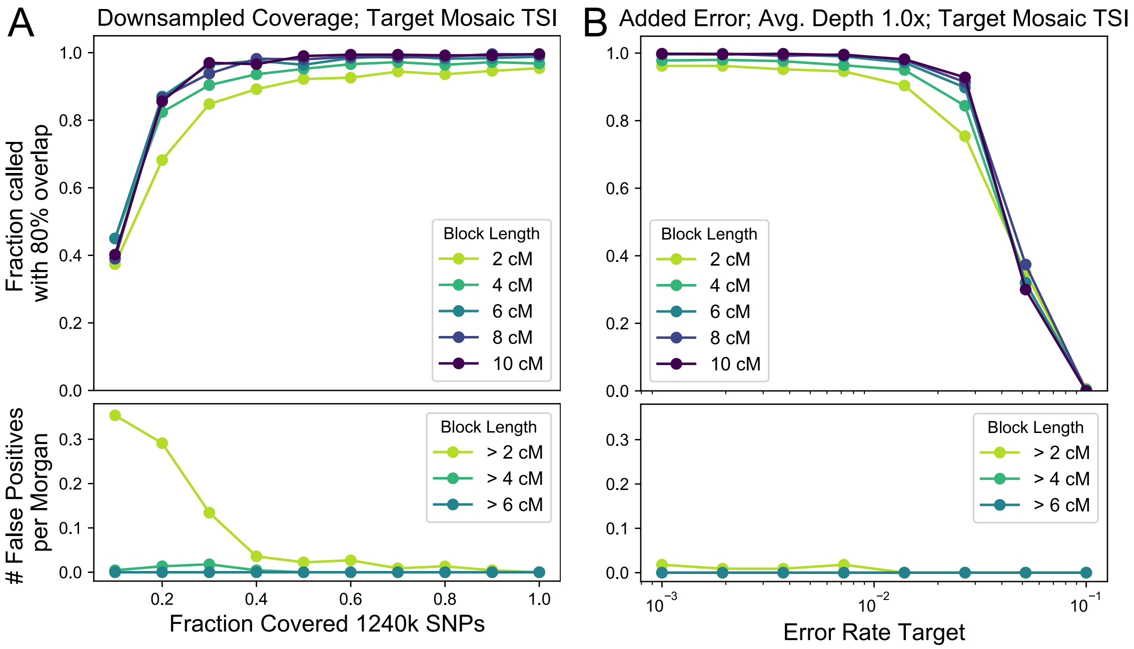

Figure S4: Performance of the method to detect ROH within mosaic individuals We analyzed 100 individual chromosomes 3 which have been copied together as mosaics from 0.25 cM stretches from TSI individuals (Tuscany) of the 1000 genomes dataset (Supplementary Note 1.7) on the 1240K sites. For each site, we then sampled one read from the diploid genotype at random, creating pseudo-haploid data. We further down-sampled to varying degrees (0.1-1.0×, Panel A), or introduced random genotype genotype errors at different rates (0.001-0.1) and applied the method with a copying error rate set to 1% (Panel B), using the 1000 genome data with the TSI haplotypes removed as reference panel (4794 haplotypes).

intuitive reason for that behavior is that for most sites in the low coverage regime ($0.1-0.6\times$ average coverage), usually at most one read per site is available, and therefore the pseudo-haploid emission model misses little information compared to models that can incorporate information from more than one read.

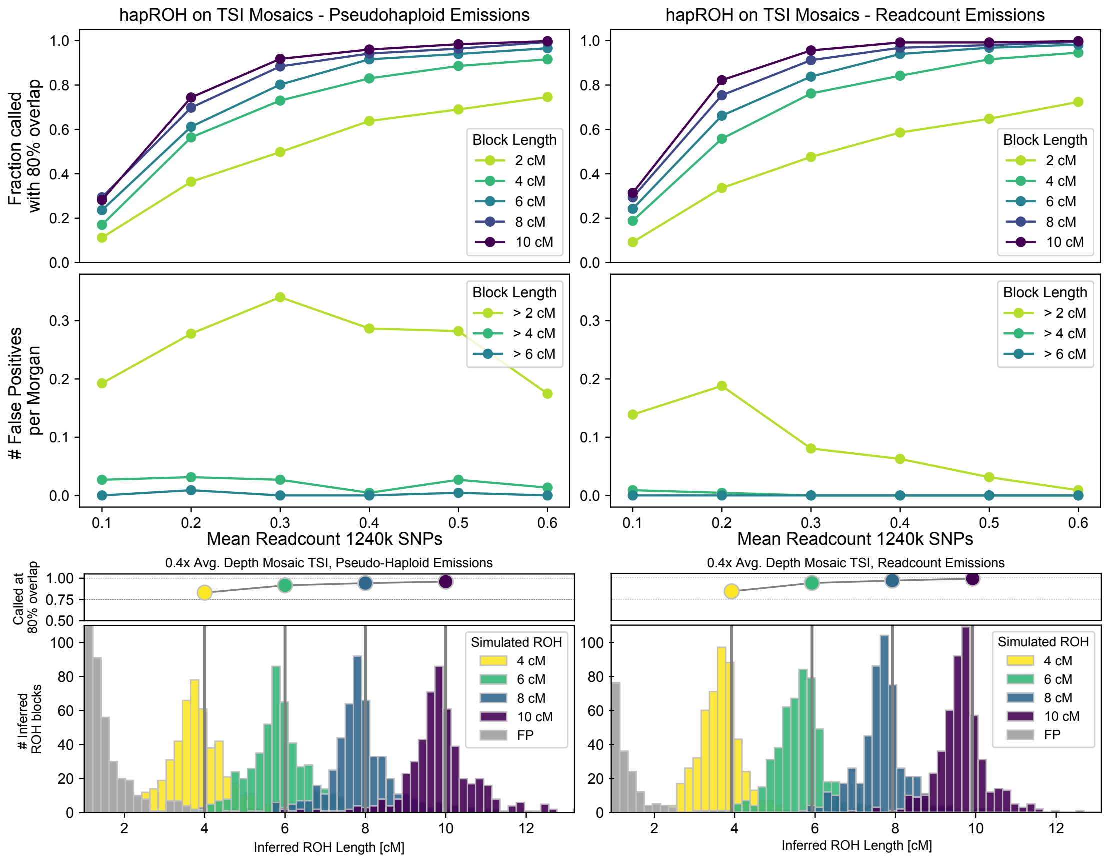

Figure S5: Performance of pseudo-haploid versus read count emission models simulated under the model. We analyzed 100 replicates of chromosomes 3 which have been copied together as mosaics from 0.25 cM stretches from TSI individuals (Tuscany) of the 1000 genomes dataset (Supplementary Note 1.7) on the 1240K sites, and for which read count data ($0.1, 0.2, \ldots, 0.6\times$ average coverage) has been generated under the Poisson model described in the text ("λ read count"). We applied the pseudo-haploid emission model (left) and full read count emission model (right). We depict performance for the full range of downsampling (first two rows) and more detailed results for $0.4\times$ average coverage (bottom row).

Supplementary Note 2.2 Reference panels with varying genetic distance

To test the impact of different coalescence time distributions to the reference panel, we applied the method on simulated mosaic individuals from various global populations when using a reference panel consisting of haplotypes from other global populations. We note that under a simple model of a clean population split, the divergence time between the target and the reference population introduces a minimum boundary for coalescence times of the reference haplotype with the reference panel, similar to a temporal separation of an ancient target from the reference panel.

First, we tested how well the method works when using a European reference panel (EUR, with TSI removed, 792 out of 1,006 haplotypes remaining) for mosaic individuals generated from several target populations of the 1000 Genomes dataset (Tab. S2 and Fig. S6A). We tested four target populations, chosen to cover a wide range of population genetic distances, and used pseudo-haploid data on 1240K SNPs, picking one allele at random at each 1240K site.

With divergence occurring tens of thousands of years ago, such as target for CHB (Han Chinese) with European reference haplotypes, 95.0% of copied-in blocks are identified with at least 80% overlap with the true ROH block. However, this behavior does not continue across all pairs of populations, we observe little power to infer ROH in mosaic individuals constructed from YRI haplotypes when using European haplotypes as reference. In this case, while some ROH blocks are still identified, only less than 10% of copied in ROH blocks are inferred with at least with 80% overlap.

Second, we tested how two other sets of reference haplotypes work for mosaic individuals generated from Tuscany (TSI) individuals, again testing our method on pseudo-haploid data on 1240K SNPs. Notably, when using East Asian reference haplotypes (EAS, 1008 haplotypes), we find that our method has limited power to call ROH at 80% overlap - with power as low as 50% for some length categories. This reduction of power is not observed when using a similar number of European haplotypes as reference for East Asian mosaics (Fig. S6A, Tab. S2). One explanation for this asymmetry could be differential drift - many models infer a smaller effective size for East Asians than in Europeans after the shared out-of-Africa bottleneck [13], plausibly causing a differential rate of loss of haplotype diversity. To test whether the effect of having a lower-diversity, non-local panel can be ameliorated, we re-ran the analysis of the mosaic TSI individuals including African reference haplotypes (AFR, 1322 haplotypes) in addition to the East Asian haplotypes. Indeed, the power to identify ROH within TSI mosaics was restored (Fig. S6B, Tab. S2). This observation indicates the benefits of using a global reference panel (as used in our empirical application).

Target	Panel	Power at 80% overlap [4cM]	Bias in Length [4cM]	Standard Deviation Length [4cM]
**TSI**	**EUR***	0.986	0.151	0.46
**CHB**	**EUR***	0.950	0.138	0.54
**CLM**	**EUR***	0.882	-0.10	0.69
**YRI**	**EUR***	0.096	-2.01	0.90
**TSI**	**EAS**	0.584	-0.74	0.93
**TSI**	**EAS+AFR**	0.934	-0.0061	0.53

Table S2: Effect of varying distance from reference panel to target. We tested the performance with mosaic individuals from Tuscany, Italy (TSI); Han Chinese from Beijing (CHB); Colombians from Medellin (CLM) and Yoruba from Ibadan (YRI) using continental reference panels from East Asia (EAS), Africa (AFR) and Europe with TSI removed (EUR*), and tested on spiked in ROH blocks of length 4 cM. As before, we define a successful inference when at least 80% of the original ROH block are inferred to be within a single inferred ROH.

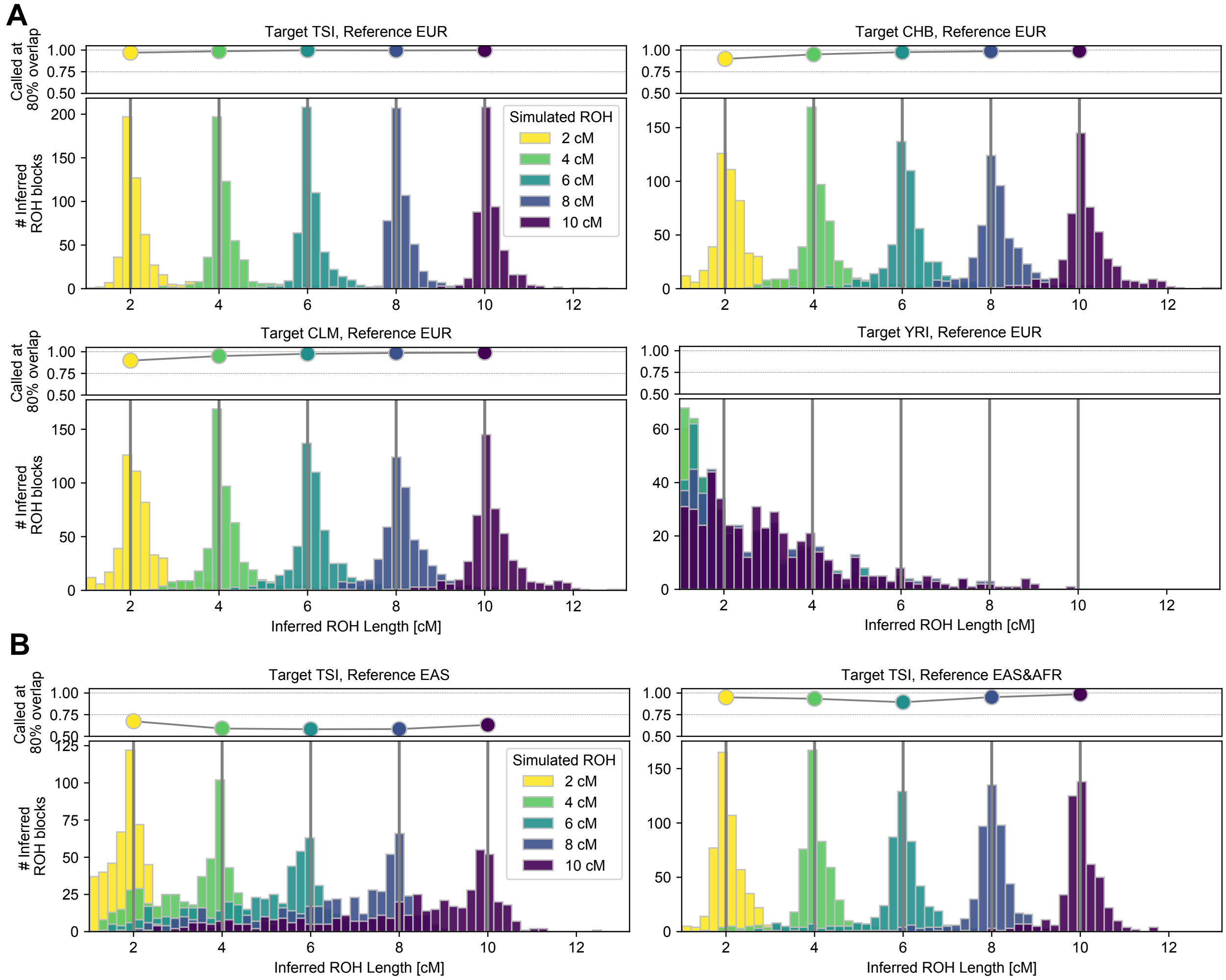

Figure S6: Effect of varying distance from reference panel to target. We tested the performance using European reference haplotypes (without TSI haplotypes) for target individuals that were simulated as mosaics of haplotypes from Tuscany, Italy (TSI); Han Chinese from Beijing (CHB); Colombians from Medellin (CLM) and Yoruba from Ibadan (YRI).

Supplementary Note 2.3 Down-sampling Ust Ishim man

High-coverage ancient DNA data provides a useful test case to assess the accuracy of ROH inference because one can compare high-coverage ROH calls to inferred ROH when down-sampling the same data. Here we analyzed a Western Siberian individual radio carbon dated to about 45,000 years before present, called "Ust Ishim man" in this way. His complete genome has been sequenced to remarkable depth (ca. 40×) from a femur bone [14], allowing for robust diploid genotype calls. Such high-coverage data allows one to robustly call ROH by directly identifying stretches that lack sites where many reads indicate heterozygosity (Fig. S7). As "Ust Ishim man" is the oldest anatomically modern human sequenced to high coverage to date (and therefore in our application), he provides us the most extreme case for testing how much temporal distance from the reference panel our method can tolerate.

We first analyzed the publicly available diploid genotype data (included in the empirical dataset we used for this study) with the diploid mode of our method, manually checking that the calls >4 cM are true gaps of heterozygous markers. We then use this ROH data as a baseline to compare to and analyzed read count data for the 1240K SNPs from Ust Ishim man (40× average read depth on the target) - using publicly available post-processed data from [12]. We first down-sampled these reads to lower coverage (0.2-40×), randomly selecting subsets of reads. We then created pseudo-haploid data for all SNPs covered (1,115,315 of the 1240K variants were covered) by choosing one remaining read at random for each SNP with at least one read covering it. We applied `hapROH` to this down-sampled pseudo-haploid data and called ROH with its pseudo-haploid emission mode.

Our results show that `hapROH` can consistently infer ROH blocks >4 cM when down-sampling to low coverage (0.5× mean depth) of the 1240K markers (Fig. S7). ROH inference seems to work reliably with as low as 0.3× coverage, with little observable bias for blocks >8 cM and a small false positive rate for blocks >4 cM (Fig. S7). We hypothesize that this is at least in part caused by the extension of shorter ROH (typical for ancient hunter gatherers) that get pushed beyond the 4 cM detection threshold.

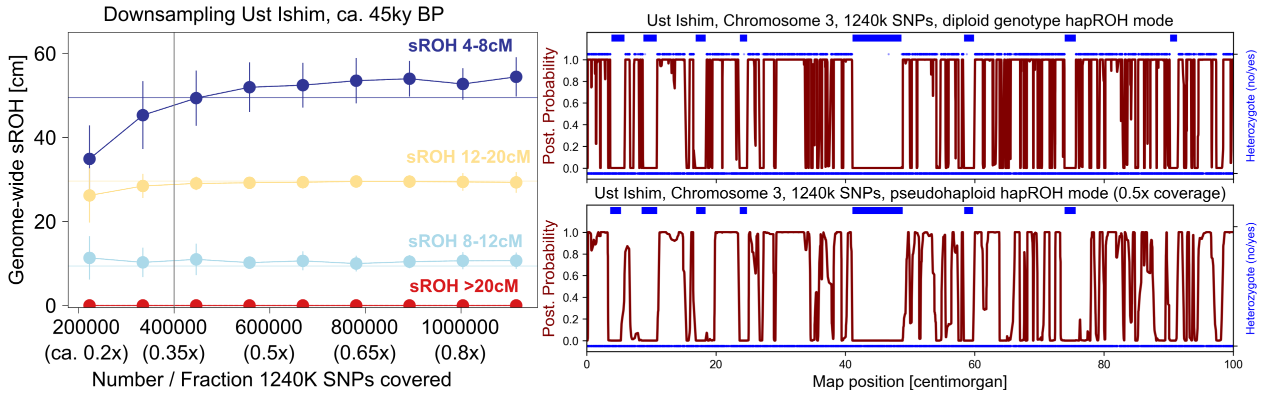

Figure S7: Properties of inferred ROH when downsampling from high coverage data on the Ust Ishim man. Left: We down-sampled read-count data from Ust Ishim man to pseudohaploid data with nine target coverages. We created 100 replicates for each of those and then applied `hapROH` in pseudohaploid mode. We depict mean and standard deviation of the inferred ROH in four length bins (4-8, 8-12, 12-20, and >20 cM). The vertical line depicts the cutoff used in the empirical data analysis. The horizontal line shows ROH using all diploid genotype data. Right: Posterior and inferred ROH for a region of Chromosome 3, when running `hapROH` in diploid genotype mode on the full diploid genotype data from Ust Ishim (top) and when running `hapROH` on pseudo-haploid data down-sampled to 0.5× coverage (bottom). We depict inferred ROH greater than 1 cM before gaps are merged as blue lines above the posterior. For the diploid genotype data, we indicate heterozygous genotypes (blue dots above the posterior) and homozygous genotypes (blue dots below the posterior).

Supplementary Note 2.4 Performance on present-day populations

We applied our method to the Human Origins (HO) dataset of 1,941 present-day humans originating from 162 global populations genotyped at autosomal SNPs [15]. These SNPs constitute a subset of the 1240K enrichment targets (≈0.6 of ≈1.24 million SNPs). Because this dataset provides diploid genotype calls, we ran our method with the diploid mode on the full diploid genotype data and called ROH >4 cM in all 1,941 individuals, using 5,008 global haplotypes from the 1000 Genomes reference panel. We manually checked inferred ROH in a number of cases, and confirmed that ROH calls correctly identify regions that lack heterozygous markers.

We also used the HO dataset to test the pseudo-haploid mode of our method. To this end, we used all HO individuals with at least one ROH longer than 12 cM identified (599 individuals) as a test set. In addition to the high quality diploid ROH calls described above, we ran the pseudo-haploid mode on these individuals, choosing one allele at random for each diploid genotype call (ca. 550,000 SNPs per individual, ranging from individuals with 537,000 to 556,000 called gentoypes). Our tests confirmed that the ROH calls from the pseudo-haploid and the diploid data closely agree for the majority of individuals (Fig. SS8), with a correlation between datasets of $r = 0.984$ when comparing ROH >8 cM (Fig. S8). A notable exception are certain Sub Saharan populations, in particular South and East African hunter gatherers, for which a substantial fraction of long ROH are not identified in the haploid data (Tab. S8).

When investigating these South and East African hunter gatherer individuals further, we noticed that the typical pattern in the inference from pseudo-haploid data is many gaps dispersed throughout ROH identified in the diploid data (e.g. Fig. S9). This pattern mirrors the one we observed when analyzing mosaic targets created from Yoruba haplotypes using an European only reference panel (Supplementary Note 1.7), pointing toward some haplotype segments not captured well by the reference panel. Indeed, it has been observed previously that hunter gatherer populations in Sub Saharan Africa possess deeply diverged ancestry [16]. Because the 1000 Genomes data only include a single population from Central, Southern and Eastern Africa (i.e. the Luhya), the possibility of missing relevant and diverged haplotypes in the reference panel yields a plausible explanation for the limited power of a method based on copying of long haplotypes.

After removing populations from Central, South and Eastern Africa, the correlation between inferred ROH from pseudo-haploid and diploid genotype data increases to $r = 0.997$. The average difference between the sum of ROH > 8 cM inferred from pseudo-haploid and diploid genotype data is -0.53 cM (the mean of the sum of ROH inferred from diploid data is 98.03 cM). Upon inspecting specific length categories, ROH calls from all length classes are highly correlated, ranging from $r = 0.925$ for ROH 4-8 cM to $r = 0.988$ for ROH longer than 20 cM (Fig. S10). These results support that using the 1000 Genome reference panel provides sufficient power to infer ROH using the pseudo-haploid mode in all but some Central, South, and Eastern Africa hunter groups in the HO data.

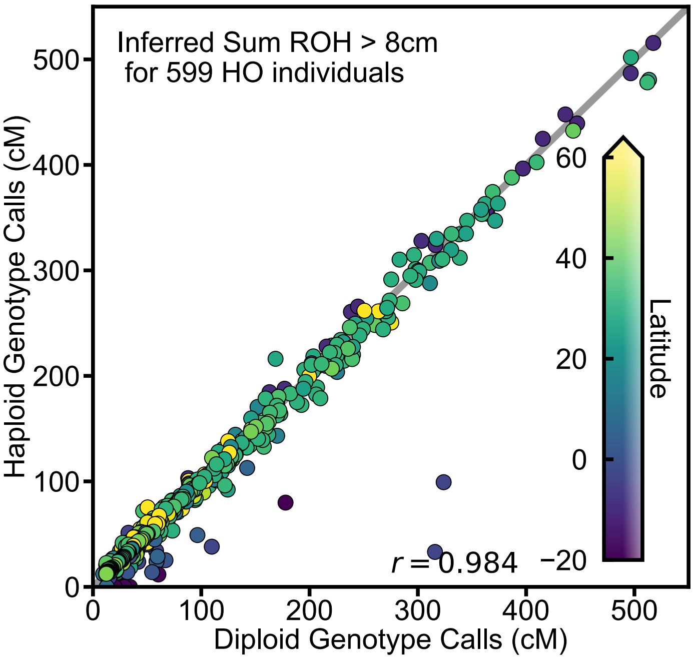

HO Population	Failed	Total
Ju_hoan_North	4	4
Hadza	3	3
Mbuti	3	4
Khomani	3	3
Biaka	2	3
Ethiopian_Jew	1	2
Somali	1	6

Figure S8 & Table S3: Comparison of diploid and pseudo-haploid ROH calls for 1,941 present-day individuals from the HO panel. Left: Comparison of ROH calls >8 cM for pseudo-haploid and diploid data for each HO individual with at least one ROH >12 cM (599 individuals). The scatter plot compares the total sum of all ROH blocks >8 cM. Right: Table summarizing individuals where more than 50% of sum ROH >8 cM are not called with pseudo-haploid data. These individuals correspond to the individuals that deviate substantially downwards from the diagonal line in the left plot.

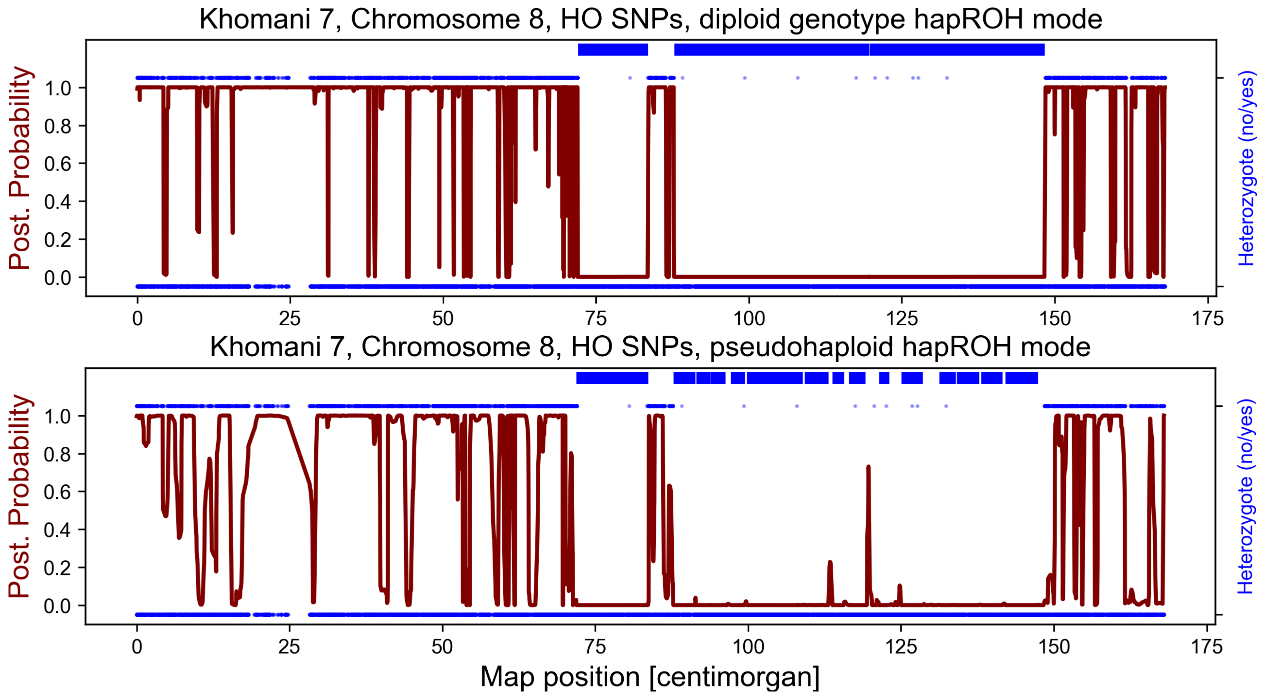

Figure S9: Comparison of diploid and pseudo-haploid ROH calls for a present-day Southern African Hunter gatherer individual. We compare the ROH calls from diploid genotype data (top) and pseudo-haploid data (bottom) from a HO African hunter gatherer in the HO origin dataset (Khomani 7). We show chromosome 8, where this individual has two long ROH on this chromosome that can be identified with high confidence in diploid genotype calls (blue dots above posterior depict heterozygous sites). The diploid mode correctly identifies these regions, whereas the pseudo-haploid mode breaks them up into multiple inferred ROH segments with gaps too big for the standard gap merging procedure.

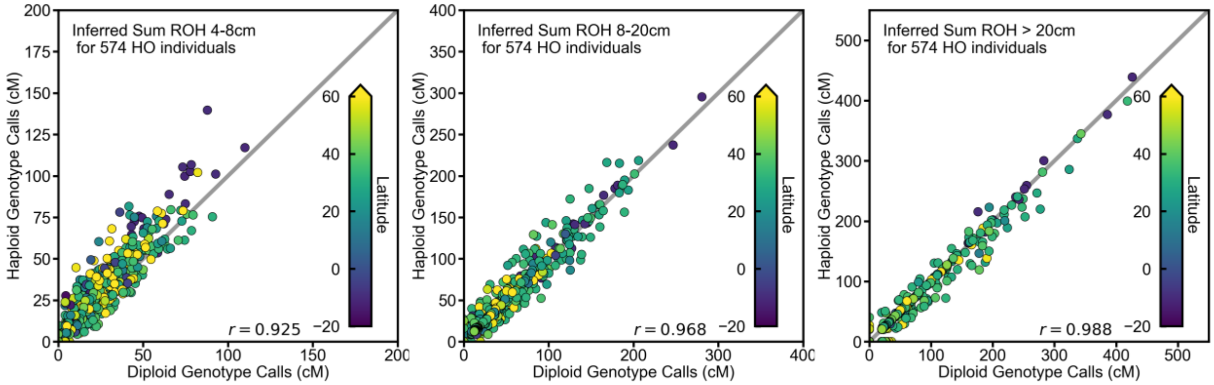

Figure S10: Comparison of diploid and pseudo-haploid ROH calls for present-day individuals from the HO panel after removing Sub Saharan populations. As in Fig. S8 we compare ROH calls for HO populations, with ROH calls from diploid genotype data (x-axis) compared to ROH calls from pseudo-haploid data (y-axis). Here we have removed the Sub Saharan populations from the panel, and show comparison for three length classes: 4-8 cM (left), 8-20 cM (middle) and >20 cM (right).

Supplementary Note 3 Comparing to other Methods

Two programs are currently widely used to identify ROH from high quality present-day data [17]. The software `PLINK` scans along the genome for windows of genotypes that lack heterozygous markers [18]. This simple but robust method uses diploid genotype calls. The second common method, `bcftools/ROH` [2] uses a HMM with two hidden states, the non-ROH state emitting homozygotes and heterozygotes, and the ROH state emitting only homozygotes, with Hardy-Weinberg proportions according to the population allele frequency at each site. It takes genotype likelihood data as input, and therefore can in principle also operate on data where coverage is too low for accurate diploid genotype calls.

We compared the performance of these methods to our method on simulated data for the 1240K array with ROH blocks spiked in, generated using the mosaic procedure detailed in Supplementary Note 1.7. For `hapROH` and `bcftools/ROH` we used allele frequencies calculated from the global reference panel used by `hapROH`. We used the default settings of each method unless specified otherwise.

We compared performance of the methods in two scenarios. First, we applied all three methods to high quality diploid genotype data, typical for SNP array data from present-day individuals. We find that all three methods have excellent power and only little bias when using diploid data (Tab. S4 and Fig. S11). However, we find that `PLINK` breaks up some long ROH when using default settings, which we did not observe when using `bcftools/ROH` or `hapROH`. This observation suggests that ROH analysis with PLINK would benefit from a post-processing step, merging short ROH gaps similar to the post-processing included by default in `hapROH` to remove spurious gaps.

The second scenario is designed to test performance on typical ancient data for which diploid genotype calls are not possible (see Fig. S12). To be able to apply `bcftools` we calculated genotype likelihoods from simulated read count data. We first generated read count data of various average coverage by using the λ-readcount model (described in Supplementary Note 2.1). To simulate genotype errors, we flipped each read to the other allele with error probability 0.001. We then calculated genotype likelihoods under the model, i.e. we assume that the probability of observing k derived out of a total of n reads for a singular SNP site given genotypes G=00, 01, or 11 is given by a binomial likelihood:

$$\Pr(\text{RC}|\text{G}) = \Pr(k, n|\text{G}) = \binom{k}{n} p^k (1-p)^{n-k} \tag{6}$$

where p denotes the probability to observe a derived read ($p = 0, 0.5,$ and 1.0 for genotypes 00,01, and 11, respectively). In these likelihood, we included a read error of 0.001 (the error used when generating the data) by modifying the read count probabilities to $p = 0.001, 0.5, 0.999$. These likelihoods were normalized and encoded in PHRED-scale in the PL field in custom output .vcfs, as required for the input of `bcftools/ROH`.

We applied `hapROH` to pseudo-haploid data generated from the same read count data, sampling one read at random at each SNP with at least one covering read. We did not run `PLINK`, as it can only operate on diploid genotype data and previous work showed that `PLINK` performs very poorly for down-sampled data, when the maximum likelihood genotype is used [19].

We find that using default settings, `hapROH` works to lower coverage than `bcftools`, in particular for ROH a few cM in length (Fig. S12). Notably, `hapROH` starts inferring false positive blocks of 2-4 cM when coverage drops below 0.5x, however the false positive rate remains negligible for blocks >4 cM (the target ROH length in our empirical analysis), and decreases for increasing coverage (Fig. S12).

We note that the performance of `bcftools` can be improved by fine-tuning the transition parameters (which is currently implemented only in a experimental setting). In principle, there is an equivalence of `bcftools/ROH` and `hapROH` HMM transition rates into and out of ROH segments; however in practice this equivalence is challenging to calibrate because of how the post-processing differs. Therefore we explored the parameter space of both methods independently. Throughout all tested parameter ranges we could not find a setting where performance was comparably to `hapROH`, indicating that using haplotype information from a phased reference panel provides a crucial advantage.

Method	Power [4cM]	Bias [4cM]	SD [4cM]	FP Rate >1cM	FP Rate > 2cM
**hapROH**	0.994	-0.0022	0.160	0.00	0.00
**bcftools/ROH**	1.000	0.0844	0.155	0.17	0.00
**PLINK**	0.986	0.1000	0.229	0.15	0.00

Table S4: Comparison of the three methods on diploid genotype data (1240K SNPS) We show performance metrics on 100 simulated Mosaic Individuals with five stretches of 4 cM, non-overlapping positions. Power is defined as ability to detect at least 80% overlap. False positive rate is calculated for 100 Chromosomes, with no ROH copied in (rate is per chromosome).

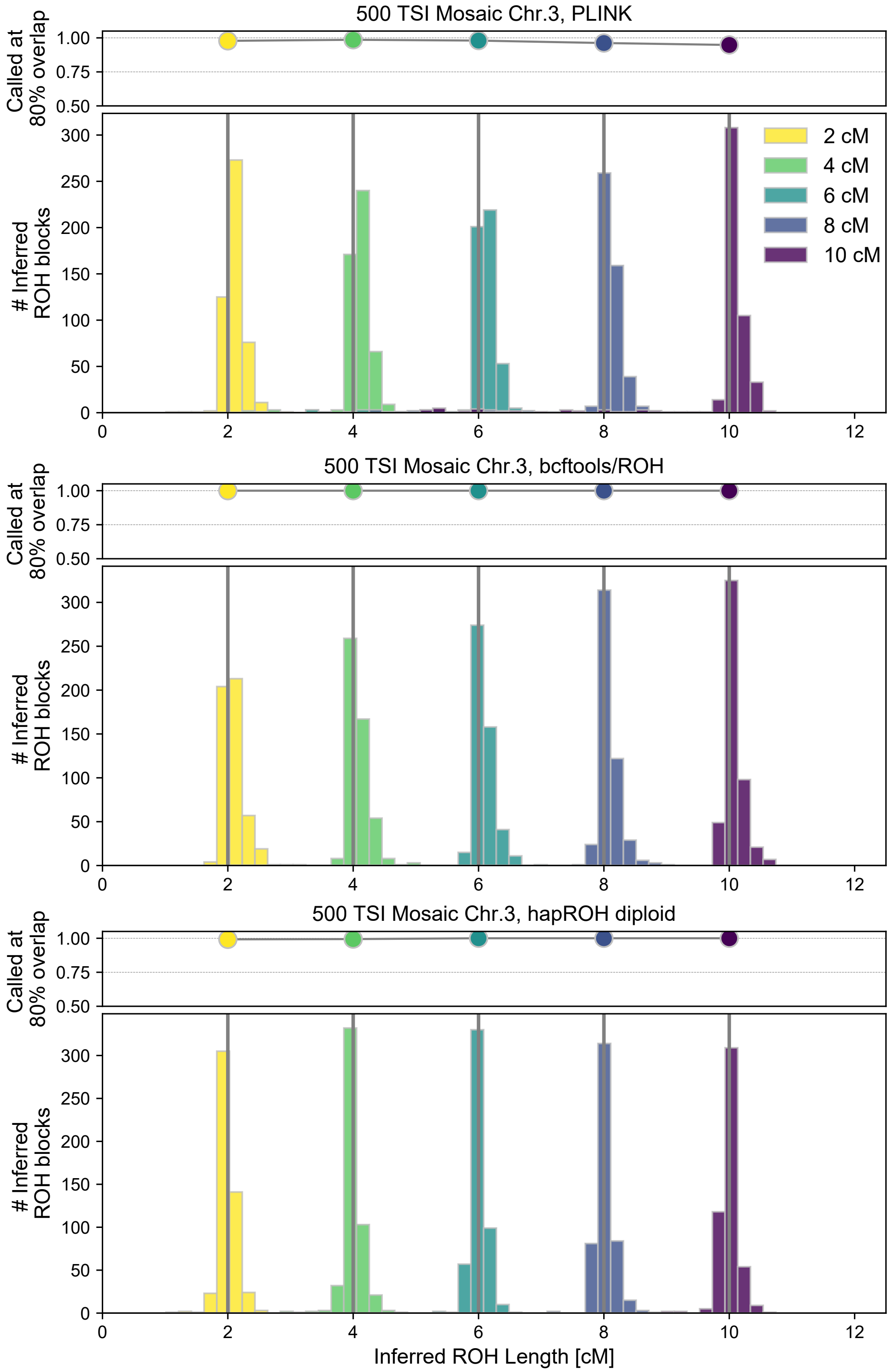

Figure S11: Comparison of the three methods to call ROH on diploid genotype data (1240K SNPS). We show performance metrics on 100 simulated Mosaic Individuals with five stretches of 2, 4, 6, 8, or 10 cM ROH copied in. Power is defined as probability to detect an ROH that overlaps at least 80% of the simulated ROH.

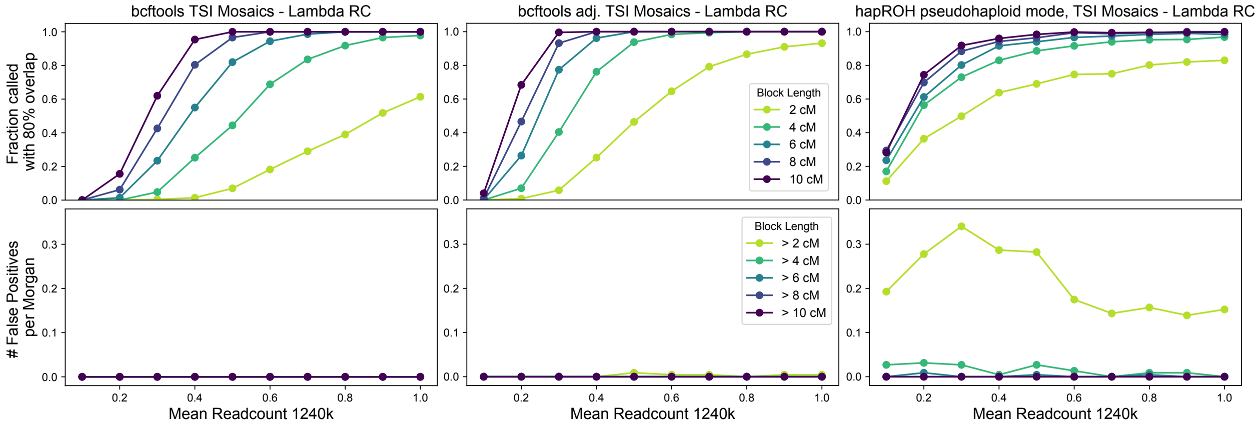

Figure S12: Comparison of `bcftools` and our method (`hapROH`) on low read count data (1240K SNPS). We show performance metrics on 100 replicates of mosaic chromosomes with five stretches of 2, 4, 6, 8, or 10 ROH copied in. We generated read count data for various mean coverage $(0.1, 0.2, \ldots, 1.0\times)$, using the λ-readcount model (described in Supplementary Note 2.1). We then applied `bcftools` using genotype likelihoods of the full read count data (calculated under the model) with standard parameters (left), parameters adjusted to best performance (middle), and `hapROH` using pseudo-haploid data from the same replicates (right).

Supplementary Note 4 Calculating Expected ROH

By design, our method identifies stretches of homozygous markers (ROH), whereas in this section we define a segment to be ended by any recombination break-point before coalescence, a definition which enables us to derive analytical approximations. Such stretches are sometimes called segments that are autozygous or homozygous by descent (HBD), as they are similar to definitions of identity-by-descent blocks between pairs of individuals [7]. Importantly, ROH and HBD segments are not equivalent, as a recent recombination event ending an HBD segment is difficult to detect if coalescent times on both sides of it are within the very recent past, resulting in segments of mostly homozygous markers on both sides of the recombination. This is akin to the "conflation" of IBD segments studied in [20]. However, for the blocks of the lengths we infer and discuss throughout this work (> 4 cM), for all but extreme levels of consanguinity, HBD and ROH can be considered identical for practical purposes, as the conflation effect decreases for long segments, and should be very rare for segments > 4 cM based on experiments with IBD segments [20].

In the following, we calculate the expected number of ROH and the expected sum of lengths for all ROH falling into given length bins, using density functions $f(x)$, i.e. the values have to be integrated $\int_{l_1}^{l_2} f(x)dx$ to give expectations within bins $[l1, l2]$. Denoting $f(x)$ as the density of the expected number of blocks of length x, the integral $\int_{l_1}^{l_2} f(x)x\,dx$ yields the expected sum of lengths of the blocks within the length bin $[l1, l2]$.

Throughout, we measure block lengths in Morgans. The first key ingredient in the derivations is the expected number $b(x|t)$ of blocks of length x caused by recombination t generations ago on a chromosome of length G Morgans. Assuming that recombination events are distributed according to a Poisson process with rate t, which is a good approximation for all but very close relatives (e.g. 1st and 2nd degree relatives, [21]), one gets:

$$b(x|t)\,dx = \underbrace{(G-x)(2t)^2\exp(-2tx)\,dx}_{(i)} + \underbrace{2(2t)\exp(-2tx)\,dx}_{(ii)},$$

where (i) describes blocks in the interior of a chromosome and (ii) from blocks delimited by one of the two chromosome boundaries. One straightforward way to derive this formula is by partitioning over all possible start sites for blocks of length x. We note that we ignored blocks extending over the whole chromosome, as in this work we are interested in shorter ROH. Combined with $\psi(t)$, the probability of coalescence t generations ago, one can then express the expected number of ROH as:

$$f(x)\,dx = \int_0^\infty b(x|t)dx\,\psi(t)\,dt. \tag{7}$$

A detailed discussion of these formulas can be found in [22].

Here, we are interested in two scenarios. First, for the offspring of full n-th cousins, where the offspring is separated by $m = 2n + 4$ meiosis (i.e. common ancestry $n + 2$ generations back), and four haplotypes are potential common ancestors:

$$\psi_n(t) = \frac{4}{2^m}\delta(n+2-t),$$

where $\delta(t)$ denotes the delta distribution. Substituting $\psi_n(t)$ into Eq. 7, we arrive at:

$$f_n(x)dx = \frac{4}{2^m}\big((G-x)m^2\exp(-xm) + 2m\exp(-xm)\big)\,dx. \tag{8}$$

Second, for constant (diploid) panmictic populations with N haploids (often denoted as twice the effective number of diploid individuals $N = 2N_e$):

$$\psi_N(t) = \exp\left(-\frac{t}{N}\right)\frac{1}{N}.$$

Applying the integral Eq. 7, we arrive at:

$$f_N(x)dx = \left(\frac{8(G-x)}{N}\frac{1}{(2x+\frac{1}{N})^3} + \frac{4}{N}\frac{1}{(2x+\frac{1}{N})^2}\right)dx.$$

This formula further simplifies to

$$f_N(x)dx = \frac{4N\,(1+2NG)}{(1+2Nx)^3}dx, \tag{9}$$

which has previously been reported in paragraph 3.1. of [23].

As outlined above, the density functions in Eq. (8) and Eq. (9) can be integrated over the interval $[l1, l2]$ to give the expected number of ROH or the expected sum of the length of all ROH falling within this interval. Here we used numerical approximations with a large number of bins (1000), which are sufficiently accurate for all practical purposes, but we note that these integrals can also be solved analytically.

We calculated the density of the expected sum of ROH blocks (Fig. S13). Our results show that offspring of parents that are close relatives, and thus have short circles in their pedigree, has most of its sum of ROH in the upper length category (20-300 cM). In contrast, loops resulting from low population sizes create bottom heavy distributions, where a substantial amount of the sum of ROH >4 cM is concentrated in ROH near the detection threshold.

For the case of a constant population size, one can use the integrand of Eq. (7) to partition the full expected sum of ROH (density) into contributions from each time point, where the integrand can be interpreted as a density of expectations per time interval. Figure S14 depicts this density for the case $2N = 500$. We observe that due to the exponential clock provided by recombination (the $\exp(-2tx)$ term), most of the total ROH of intermediate length classes (depicted for 4, 8, 12, and 20 cM) originate from recent timescales. For short blocks of length 4 cM, there is a substantial contribution from up to 100 generations ago (with less than 1% expected contribution from beyond that), whereas for longer blocks 12 cM substantial contributions from only up to 20 generations ago arise. Also note that blocks from certain generations are more likely to result in the required length, therefore initially the density goes up when going back in time. All these qualitative patterns will in fact hold for all but extreme scenarios of demography (producing exponentially growing coalescent rates back in time) that would counteract the exponential recombination clock, analogously to IBD blocks between individuals [22].

To validate the analytical formulas, we simulated ROH in panmictic populations of sizes $2N = 500, 1,000, 2,000,$ and $4,000$, using the software `msprime` [24]. We simulate ROH on

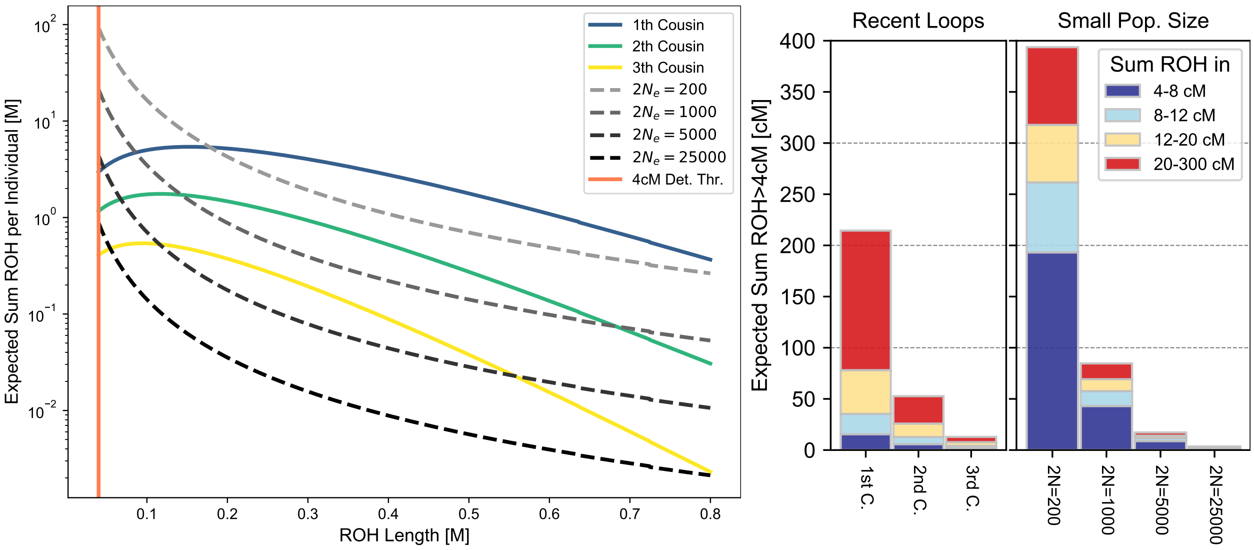

Figure S13: Density of expected sum of ROH per individual We calculated the density of the sum of ROH per length bin for parents being full cousins of degree $1, 2, 3$ and effective population sizes $200, 1000, 5000$ by multiplying the expected number of ROH blocks $f(x)$ from Eq. (8) and Eq. (9) with the length x. Integrating $\int_{l_1}^{l_2} f(x)x\,dx$ would yield the expected sum of block lengths within the length bin $[l_1, l_2]$. Left: Expected densities. The vertical line depicts the detection cut-off we applied in our analysis of the ancient data. Right: Integral of expected densities over bins used in the empirical analysis (4-8, 8-12, 12-20, >20 cM).

all autosomes, each chromosome in a separate run. For chromosome lengths, we used the map difference between the first and last 1240K SNP on each autosome, both in analytical formulas and the simulations. We defined ROH as regions delimited by two recombination events in the full ARG when simulating two haplotypes. When binning the ROH values into length bins as used in the main paper (4-8, 8-12, 12-20, and >20 cM), the average values over replicate individuals within this bins agree closely with the average of the simulated values (Fig. S15).

Similarly, we simulated the offspring of cousins of various degrees, which are described in detail in Supplementary Note 5. Again, the simulated values (when averaged over a large number of replicates) and analytical values are in close agreement (Fig. S16), validating the formulas derived here.

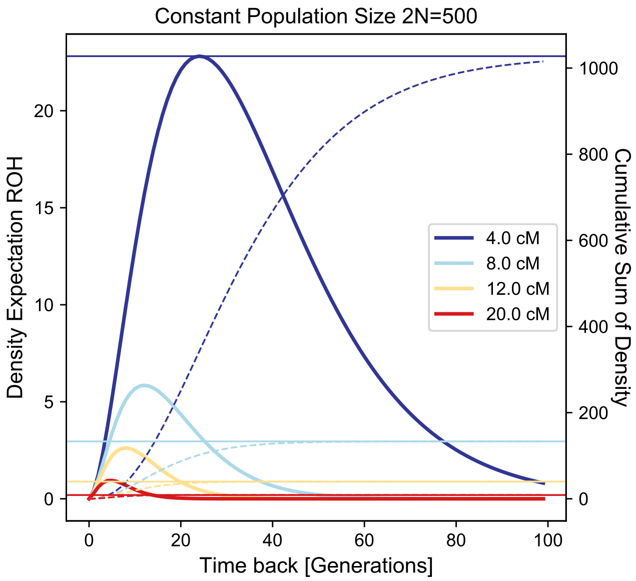

Figure S14: Timescales of ROH sharing for constant population size. The density of expected ROH blocks with respect to time for a constant panmictic population of size $2N = 500$, depicted for block lengths 4, 8, 12, and 20 cM. We show the cumulative sum of these expectations (dotted curves, y axis labels right axis) when summed over time and also the analytical integral over all times from Eq. (9) (horizontal lines). Calculations were done with chromosome lengths of the human autosomes, and then summing the contribution from each chromosome.

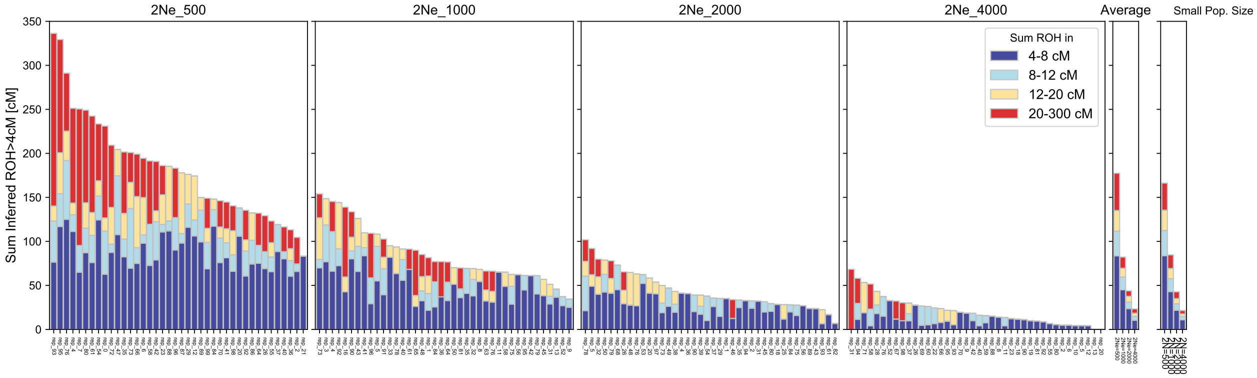

Figure S15: Simulated ROH for four population sizes. We visualize the simulated ROH distribution on all autosomes for $2N_e = 500, 1,000, 2,000, 4,000$. Each bar represents ROH of one simulated individual (40 independent replicates per population size). The panel denoted "Average" gives the empirical average for each of these groups, and the panel denoted "Small Pop. Size" gives the analytical average calculated from formula, Equation (9).

Supplementary Note 5 Simulating ROH Lengths

To gain insight into the length distribution of ROH blocks for a given degree of parental relatedness, one can calculate expected numbers of blocks falling into certain length classes (see Supplementary Note 4). However, these calculations do not yield insight into the variance of the distribution, and also rely on the assumption that recombination can be modelled as a Poisson process (when genomic distances are measured in Morgan) and do not incorporate the biological process of recombination interference (i.e. recombination events are less clustered than expected) as well as sex-specific recombination maps. For distant relatives beyond second degree these model violations have only minimal impact [21], but this leaves the possibility that this process can significantly influence ROH patterns when an individual's parents are close relatives.

For these reasons, we utilized the recently developed method ped-sim (v1.0.6) to simulate shared blocks of genome between close relatives [21] to gain insight into the length distribution of ROH blocks. Importantly, this simulation engine can incorporate both sex-specific recombination maps as well as recombination interference.

We simulated 1000 full individuals each, using the sex-specific genomic map of [25], and simulating all autosomes. We then cluster individual ROH into bins of various lengths, as done in the empirical analysis. Our simulations demonstrated that ROH sharing among 1st cousin offspring of otherwise outbred individuals ranges from ca. 50-500 cM for 1000 simulated first-cousin-offspring, with a mean expected value of 1/16th of the autosomal genome, 225 cM. Our results also show that the rate of ROH longer than 20 cM drops quickly with the increasing degree of parental relatedness (Fig. S16). When simulating 1000 replicates for each parental relatedness scenario, for offspring of parents who are (full) first cousins, 97.7% have at least one ROH longer than 20 cM (95% binomial CI: 96.6-98.5%), for second cousins, this fraction drops to 57.1% (53.9-60.2%), and for offspring of fifth cousin it is only 0.2% (0.02-0.72%) (Tab. S5).

Based on these simulations, we mark individuals as being potential offspring of very closely related parents if the sum of ROH >20 cM exceeds 50 cM. Ca. 88% of all first cousins offspring and 20% of all second cousin offspring pass this threshold. However less than 1% of third and less than 0.1% of offspring of parents fourth or further, fall above the threshold. Even if power to detect long ROH in this length class would be only 50% (a value far below the power estimates from our simulation and down-sampling experiments), one would still expect to detect ca. 60% of all first cousins in the dataset (Fig. S17).

Parents being...	Replicates	4-8 cM	8-12 cM	12-20 cM	>20 cM
1st_cousin	1000	913	848	939	977
2nd_cousin	1000	625	476	557	571
3rd_cousin	1000	289	172	227	142
4th_cousin	1000	107	68	65	23
5th_cousin	1000	23	12	20	2

Parents being	sum(ROH >20 cM) >50	sum(ROH >20 cM) >100
1st_cousin	883	602
2nd_cousin	201	27
3rd_cousin	8	0
4th_cousin	0	0
5th_cousin	1	0

Table S5: Number of simulated individuals with ROH within a given length class. We simulated 1000 individuals for each class of parental relatedness. The upper table gives the number of individuals which have at least one ROH in a given length class on any of their autosomes (each ROH length class is one column), the lower table the number of individuals with at least a certain amount of ROH longer than > 20 (when summing over all such blocks).

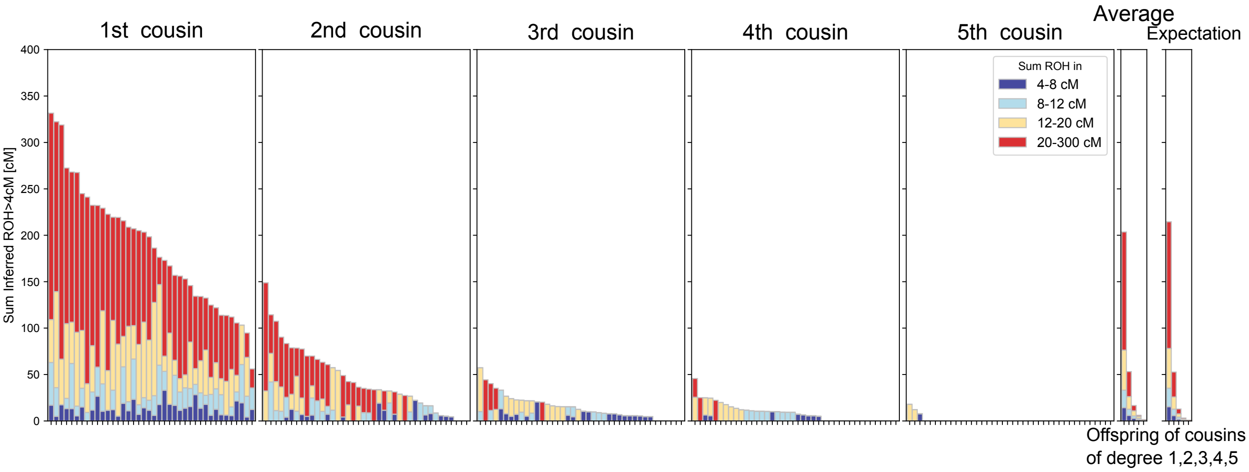

Figure S16: Simulated ROH in offspring of cousins of various degrees of relatedness. We used the software `pedsim` to simulate ROH given various degrees of parental relatedness on all autosomes. The software modelled both recombination interference and sex-specific genetic maps. Each bar visualizes one individual and we color-code the sum of ROH in distinct length classes. For each parental degree of relatedness (1st to 5th full cousins, i.e. relatedness via both a male and female shared ancestor) we show 40 replicates. The panel denoted "Average" shows the empirical average for each ROH length bin. The panel denoted "Expectation" shows the corresponding expectation calculated from formula Eq. (8).

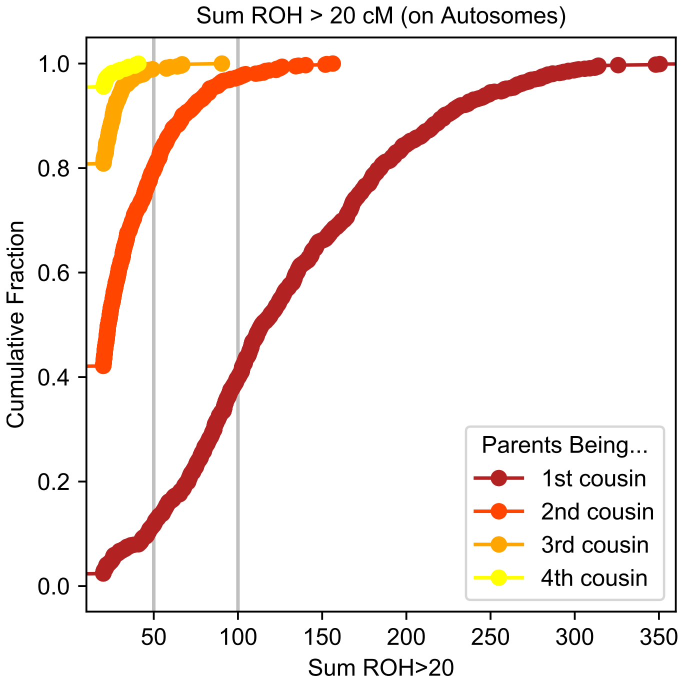

Figure S17: Distribution of ROH > 20cM in offspring of cousins of various degrees of relatedness We show the cumulative distribution of the sum ROH> 20cM for 1000 simulated individuals each for 1st to 4th degree. The gray vertical bars depict 50 and 100 cM, which are used as threshold in the main manuscript.

Supplementary Note 6 Properties of SNP Set

We developed and applied hapROH primarily focusing on a set of 1.24 million SNPs across the human genome widely used as capture targets in human ancient DNA studies (the "1240K" SNP panel). This set of SNPs was designed to intersect with the Affymetrix Human Origins array and Illumina 610-Quad array [8]. While considerable variation in the density of the 1240K SNPs along the genome exists (see Fig. S18), there is no large region devoid of any SNPs. Thus our genome-wide analysis of relatively long ROH should not suffer from systematic biases caused by absence of SNPs in some regions. For analysis of modern data we used the Human Origin SNPs which are a strict subset of ca. 550,000 SNPs of this 1240K capture array.

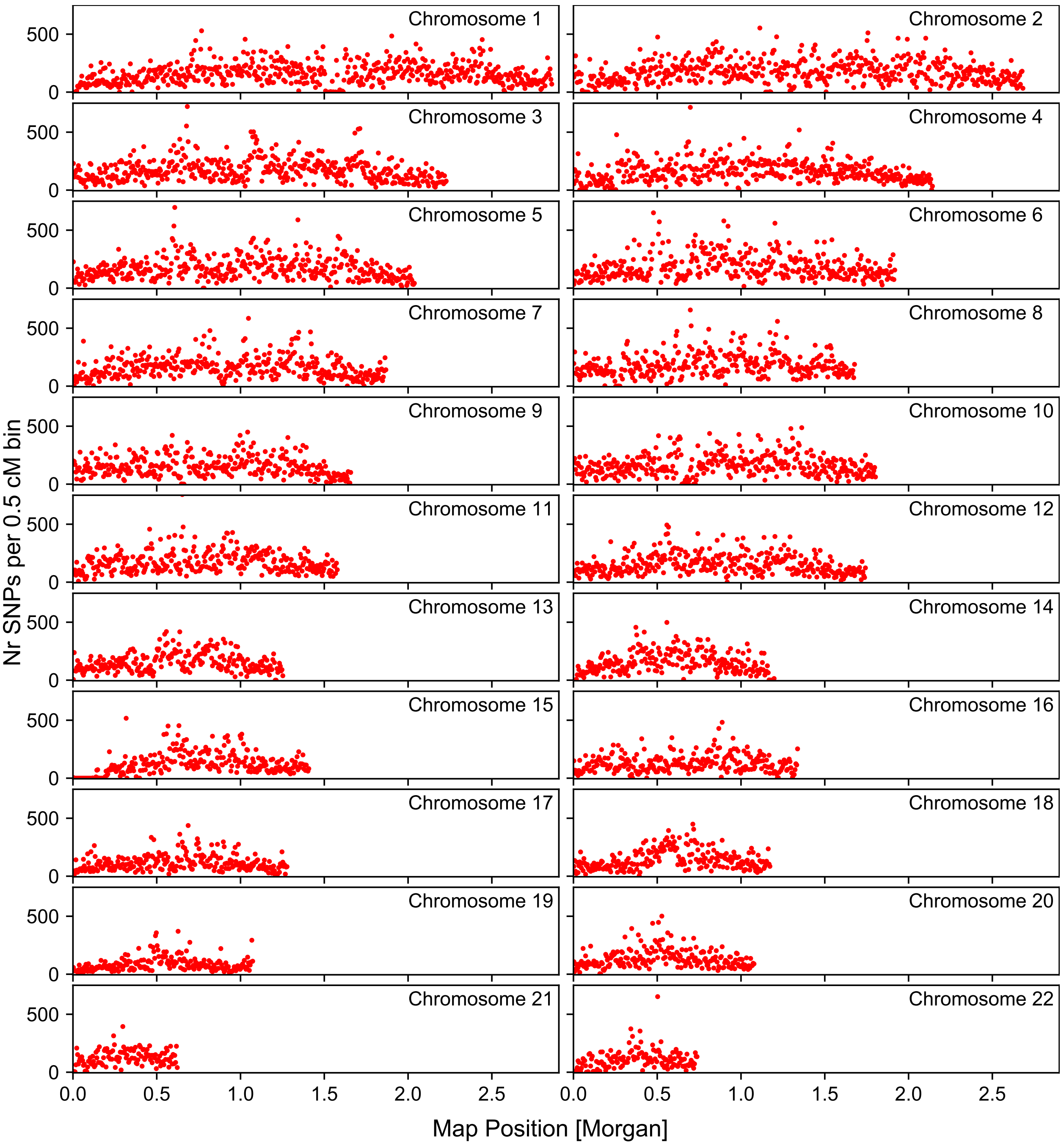

Figure S18: Distribution of 1240K SNPs along the genome. We depict the number of 1240k SNPs along each chromosome in 0.5 cM bins (red dots).

Supplementary Note 7 Properties of ancient Data Set

As outlined in the methods, the bulk of our global ancient DNA dataset originates from a curated dataset of published ancient DNA (released on March 1, 2020, v42), available via https://reich.hms.harvard.edu. This release provides ancient DNA data in pseudo-haploid format with genotypes for the 1240K SNP set. It also contains individuals with whole genome sequenced data available, which had been down-sampled to this set of over a million SNPs. Here, we visualize three key statistics of this data set, as reported in the meta-file: 1) Age Distribution 2) Average Coverage and 3) Estimated autosomal contamination, available for males based on hemizygous X chromosomes (Fig. S19).

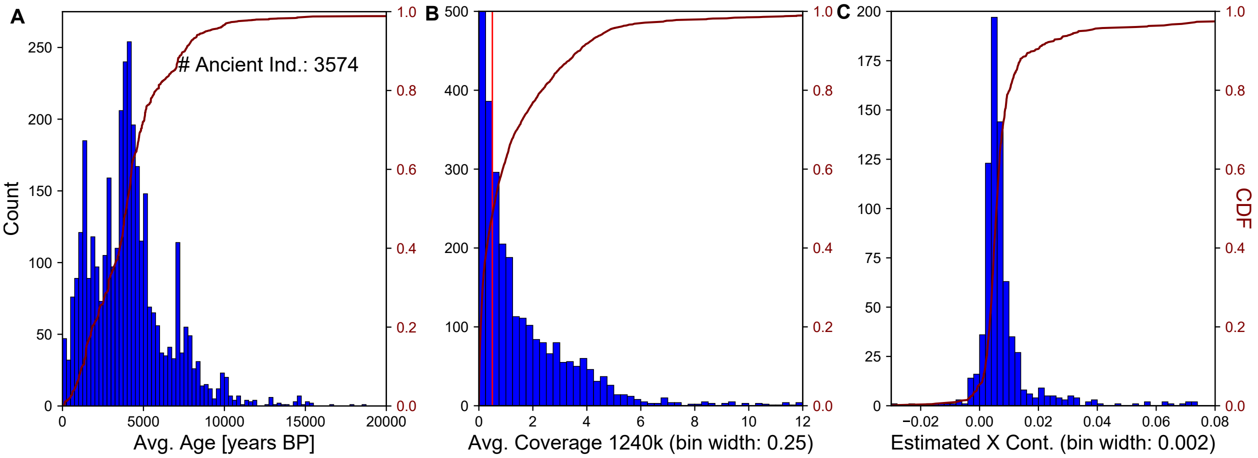

Figure S19: Data Details of ancient Individuals. We depict key properties of publicly available ancient individuals downloaded from https://reich.hms.harvard.edu (v42). For each ancient individual we kept the record with the highest coverage (several have been genotyped multiple times). We depict histogram to visualize distribution of reported data properties of ancient individuals. Panel A: Age of each individual (mean of radio carbon dates where available, mean of context dates otherwise). Panel B: Mean Coverage on autosomal SNPs (1240K polymorphisms). Panel C: Mean reported error estimates (X contamination estimates ANGSD [26], MOM point estimator, which can be negative due to estimation uncertainty).

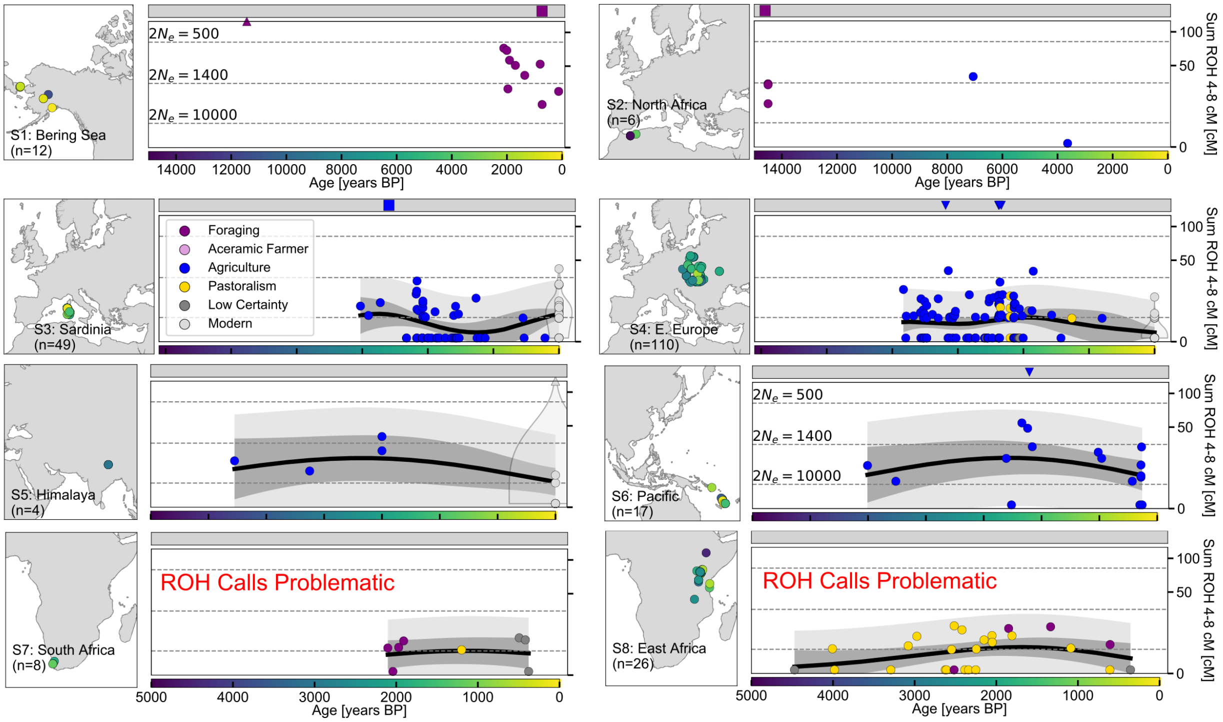

Figure S20: Time transects of ROH in eight additional regions. Each circle in the main panels depicts $\text{sROH}_{[4,8]}$ for one ancient individual. We plot individuals grouped by geography. We also show mean estimates calculated from a Gaussian Process model (solid black lines, see `Methods`), and 95% empirical confidence intervals for individuals (light gray) and for the estimated mean (dark gray). Horizontal lines depict the theoretical expectations for sum of ROH blocks 4-8 cM for panmictic population of different sizes, calculated using analytical formulas (Supplementary Note 4). In the gray bar at the top of each panel, we indicate individuals with $\text{sROH}_{>20}$ more than 100 cM (squares) and 50 cM (downward triangles). Where available, we also show ROH in present-day individuals (light-gray points for each individual, violin plot for density estimate). As `hapROH` could not recover a substantial fraction of long ROH for certain Sub-Saharan ancestries (see Supplementary Note 2.4 for details) we warn that the power to call ROH is likely also low for ancient Sub-Saharan African individuals.

Supplementary References

[1] Li, N. & Stephens, M. Modeling linkage disequilibrium and identifying recombination hotspots using single-nucleotide polymorphism data. *Genetics* **165**, 2213–2233 (2003).

[2] Narasimhan, V. *et al.* BCFtools/RoH: a hidden Markov model approach for detecting autozygosity from next-generation sequencing data. *Bioinformatics* **32**, 1749–1751 (2016).

[3] Racimo, F., Renaud, G. & Slatkin, M. Joint estimation of contamination, error and demography for nuclear dna from ancient humans. *PLoS Genetics* **12** (2016).

[4] Prüfer, K. snpAD: An ancient DNA genotype caller. *Bioinformatics* **34**, 4165–4171 (2018).

[5] Durbin, R., Eddy, S. R., Krogh, A. & Mitchison, G. *Biological Sequence Analysis: Probabilistic Models of Proteins and Nucleic Acids* (Cambridge University Press, 1998).

[6] Price, A. L. *et al.* Sensitive detection of chromosomal segments of distinct ancestry in admixed populations. *PLoS Genetics* **5** (2009).

[7] Ralph, P. & Coop, G. The geography of recent genetic ancestry across europe. *PLoS Biology* **11**, e1001555 (2013).

[8] Fu, Q. *et al.* An early modern human from Romania with a recent Neanderthal ancestor. *Nature* **524**, 216 (2015).

[9] Briggs, A. W. *et al.* Patterns of damage in genomic DNA sequences from a Neandertal. *Proceedings of the National Academy of Sciences* **104**, 14616–14621 (2007).

[10] Glenn, T. C. Field guide to next-generation DNA sequencers. *Molecular ecology resources* **11**, 759–769 (2011).

[11] Browning, S. R. & Browning, B. L. Accurate non-parametric estimation of recent effective population size from segments of identity by descent. *American Journal of Human Genetics* **97**, 404–418 (2015).

[12] Marcus, J. H. *et al.* Genetic history from the Middle Neolithic to present on the Mediterranean island of Sardinia. *Nature Communications* **11**, 1–14 (2020).

[13] Keinan, A., Mullikin, J. C., Patterson, N. & Reich, D. Measurement of the human allele frequency spectrum demonstrates greater genetic drift in East Asians than in Europeans. *Nature genetics* **39**, 1251–1255 (2007).

[14] Fu, Q. *et al.* Genome sequence of a 45,000-year-old modern human from western Siberia. *Nature* **514**, 445–449 (2014).

[15] Lazaridis, I. *et al.* Ancient human genomes suggest three ancestral populations for present-day Europeans. *Nature* **513**, 409–413 (2014).

[16] Schlebusch, C. M. *et al.* Genomic variation in seven Khoe-San groups reveals adaptation and complex African history. *Science* **338**, 374–379 (2012).

[17] Ceballos, F. C., Joshi, P. K., Clark, D. W., Ramsay, M. & Wilson, J. F. Runs of homozygosity: windows into population history and trait architecture. *Nature Reviews Genetics* **19**, 220 (2018).

[18] Purcell, S. *et al.* PLINK: a tool set for whole-genome association and population-based linkage analyses. *American Journal of Human Genetics* **81**, 559–575 (2007).

[19] Renaud, G., Hanghøj, K., Korneliussen, T. S., Willerslev, E. & Orlando, L. Joint estimates of heterozygosity and runs of homozygosity for modern and ancient samples. *Genetics* genetics–302057 (2019).

[20] Chiang, C. W., Ralph, P. & Novembre, J. Conflation of short identity-by-descent segments bias their inferred length distribution. *G3: Genes— Genomes— Genetics* **6**, 1287–1296 (2016).

[21] Caballero, M. *et al.* Crossover interference and sex-specific genetic maps shape identical by descent sharing in close relatives. *PLoS Genetics* **15**, e1007979 (2019).

[22] Ringbauer, H., Coop, G. & Barton, N. H. Inferring recent demography from isolation by distance of long shared sequence blocks. *Genetics* **205**, 1335–1351 (2017).

[23] Carmi, S., Wilton, P. R., Wakeley, J. & Pe'er, I. A renewal theory approach to ibd sharing. *Theoretical Population Biology* **97**, 35–48 (2014).

[24] Kelleher, J., Etheridge, A. M. & McVean, G. Efficient coalescent simulation and genealogical analysis for large sample sizes. *PLoS Computational Biology* **12** (2016).

[25] Bhérer, C., Campbell, C. L. & Auton, A. Refined genetic maps reveal sexual dimorphism in human meiotic recombination at multiple scales. *Nature Communications* **8**, 1–9 (2017).

[26] Korneliussen, T. S., Albrechtsen, A. & Nielsen, R. ANGSD: analysis of next generation sequencing data. *BMC Bioinformatics* **15**, 356 (2014).
