# Supplementary material for: Parental relatedness through time revealed by runs of homozygosity in ancient DNA
Source: Nat Commun. 2021 Sep 14;12:5425. doi: 10.1038/s41467-021-25289-w (PMC8440622; doi:10.1038/s41467-021-25289-w)
Supplement: Supplementary file 3 — Description of Additional Supplementary Files [file 41467_2021_25289_MOESM3_ESM.pdf]

Description of Additional Supplementary Files

File Name: Supplementary Data 1
Description: Sheet A: Individual ROH

This table lists the individul ROH results and relevant meta data for all individuals analyzed in this study (including age, longitude, latitude, region assignment, original study and subsistence strategy assignment). Each row is one individual.

Sheet B: Primary References

Full list of references and DOIs to the 103 primary publications of ancient DNA data screened for ROH.

Sheet C: Figure Data

A table linke each Figure, Table, Supp. Figure and Supp. Table to the necearry data, and code to generate this data.

Sheet D: Region Definitions

A table listing the geographic corner points (in decimal latitude and longitude) of the regions shown in Fig. 1.
